# Cytoplasmic and nuclear extracellular signal-regulated kinases are necessary for *Campylobacter jejuni* infection

**DOI:** 10.3389/fmicb.2026.1854036

**Published:** 2026-06-08

**Authors:** Megan C. Dines, Prabhat K. Talukdar, Sawyer D. Hicks, Claire A. McKnight, Lisa M. Gloss, Eric A. Shelden, Michael E. Konkel

**Affiliations:** 1 School of Molecular Biosciences, College of Veterinary Medicine, Washington State University, Pullman, WA, United States; 2 Honors College, Washington State University, Pullman, WA, United States

**Keywords:** bacterial effector, bacterial host cell invasion, ERK nuclear translocation, interleukin-8, MAP kinase-docking site

## Abstract

**Introduction:**

Acute *Campylobacter jejuni* infection relies on effector proteins delivered from the flagellar apparatus to gut epithelial cells. While the *C. jejuni Campylobacter* invasion antigen D (CiaD) secreted protein has been linked to extracellular signal-regulated kinase (ERK) activation, the contribution of ERK in establishing acute bacterial infection has not been fully explored. We hypothesized that subcellular ERK could play distinct roles in *C. jejuni* proinflammatory cytokine production and bacterial invasion. The objective of this study was to determine the contribution of cytoplasmic and nuclear ERKs in response to *C. jejuni* infection with a wild-type isolate and effector mutants.

**Methods:**

Epithelial cells were infected with *C. jejuni* in the presence of focal adhesion inhibitors that prevent extracellular signal-regulated kinases 1 and 2 (ERK1/2) activation and a peptide that disrupts ERK nuclear translocation. We measured markers of *C. jejuni* infection, including cell invasion and interleukin-8 (IL-8) cytokine secretion by gentamicin-protection assay and ELISA, respectively.

**Results:**

Our results demonstrate that inhibitors of ERK1/2 activation drastically reduce both the *C. jejuni* internalization and IL-8 secretion. Moreover, ERK1/2 activation was dependent upon its association with the focal adhesion protein paxillin, which is mediated by the *C. jejuni* CiaD effector. Blocking ERK nuclear translocation with a phosphomimetic peptide results in a significant reduction in IL-8 secretion, and surprisingly, bacterial invasion.

**Discussion:**

We conclude that nuclear ERK is required for *C. jejuni*-induced IL-8 secretion and that both cytosolic and nuclear ERK are necessary for *C. jejuni* maximal cell invasion. Targeting host ERK signaling represents a promising therapeutic strategy to reduce the severity of *C. jejuni*-mediated enteritis.

## 1 Introduction

Microbial pathogens have developed various strategies to survive in a host, including manipulating host cell gene expression and signaling pathways to subvert host cell functions. The extracellular signal-regulated kinases 1 and 2 (ERK1/2) are ideal targets, as these two proteins are essential for many cellular processes, including cell growth, proliferation, and survival pathways. Some pathogens manipulate ERK1/2 to promote cell invasion and survival, including *Salmonella enterica* and *Shigella flexneri* (Romero et al., 2011; Hume et al., 2017). Other pathogens, such as *Campylobacter jejuni, Chlamydia trachomatis, Mycobacterium tuberculosis, Yersinia* species, and *Vibrio parahaemolyticus*, dysregulate ERK1/2 signaling pathways to evade or potentiate the immune response (Boland and Cornelis, 1998; Buchholz and Stephens, 2007; Eucker et al., 2014; Li et al., 2015; Cao et al., 2025). The specific mechanisms by which bacterial pathogens modulate ERK1/2 activity vary. For example, *Shigella* invasion of epithelial cells requires ERK1/2, which, when phosphorylated, alters filopodia dynamics and enables bacterial capture (Romero et al., 2011). In contrast, *M. tuberculosis* delivers a tyrosine phosphatase (mPTPB) to the cytoplasm of macrophages that decreases ERK1/2 phosphorylation, blocking the production of IL-6 and activating the Akt pathway to enhance cell survival (Zhou et al., 2010). However, it is not fully understood how the subcellular localization of ERK potentiates acute *C. jejuni* infection.

*C. jejuni* is one of the leading causes of bacterial gastroenteritis in the United States and worldwide. An estimated 1.5 million people are infected with *C. jejuni* each year in the United States, generating an economic cost of $11.3 billion (Hoffmann et al., 2025). Infection with *C. jejuni* is frequently associated with eating foods cross-contaminated with raw or undercooked poultry. The most common symptoms are diarrhea, often with bloody stools, abdominal pain, malaise, and vomiting. Individuals with acute infections may face a range of post-infectious complications, such as the debilitating Guillain-Barré syndrome (GBS), irritable bowel syndrome (IBS), and reactive arthritis. In low and middle-income countries, the impact is even more dire, as infections can lead to malnourishment and stunted growth, affecting an individual's educational attainment due to cognitive delays and an increased risk of chronic diseases later in life. Acute *C. jejuni* disease is associated with bacterial invasion of the gut epithelial cells and the pathogen-induced inflammatory response (Negretti et al., 2020).

The genus *Campylobacter* belongs to the class ε-proteobacteria, whereas the bacterial species *Salmonella, Escherichia*, and *Shigella* are in the class γ-proteobacteria. Although sequencing the *Campylobacter* genome was expected to provide crucial information (e.g., a list of putative virulence genes, niche-specific genes, and nucleotide variations that affect pathogenicity), the genes whose products contribute to *C. jejuni* disease have remained elusive (Duong and Konkel, 2009). Notably, *C. jejuni* exhibits an altered synthetic response when co-cultured with epithelial cells, and a subset of the newly synthesized proteins is secreted from the bacterial flagellar apparatus (Konkel and Cieplak, 1992; Konkel et al., 1993, 1999). A novel genetic screen was then used to identify a candidate list of *C. jejuni* secreted proteins, leading to the identification of *Campylobacter* invasion antigen D (CiaD) (Christensen et al., 2009). Secretion and delivery assays performed with a *ciaD* mutant harboring a plasmid encoding CiaD fused to the adenylate cyclase domain (ACD) of *Bordetella pertussis* confirmed that CiaD is secreted from the bacterium and delivered to the cytosol of host cells (Samuelson et al., 2013). Subsequently, CiaD was found to bind to the host cell protein IQ-motif containing GTPase activating protein 1 (IQGAP1) to modify Rac1 activation, promoting actin reorganization and bacterial uptake (Negretti et al., 2021). IQGAP1 is a scaffold protein that coordinates actin reorganization. Relevant to this finding is that *S. enterica* serotype Typhimurium usurps IQGAP1 to manipulate Rac1 activity and the MAPK/ERK signaling pathway (Kim et al., 2011).

*C. jejuni* infection results in a proinflammatory response, as judged by the clinical presentation of disease (Blaser et al., 1979; Black et al., 1988), and the detection of interleukin-8 (IL-8) mRNA in the stool (Bennett et al., 2010). Presumably, the onset of a robust immune response promotes infection at the early stages by enabling colonization of the host's gut. *In vitro* studies have revealed that *C. jejuni* is a potent stimulator of secretion of IL-8 from a variety of cultured cells, including INT 407, T84, and IPEC-1 cells (Hickey et al., 1999; Watson and Galan, 2005; Zheng et al., 2008; Murphy et al., 2011; Eucker et al., 2014). Additional studies revealed that *C. jejuni* requires *de novo* protein synthesis to stimulate IL-8 secretion from cells and that the ERK kinase signaling pathway is essential for IL-8 production (Watson and Galan, 2005; Eucker et al., 2014). In contrast to some bacteria, the flagellin of α- and ε-Proteobacteria, including *C. jejuni*, lacks a specific Toll-like receptor 5 (TLR-5) sequence and does not activate TLR-5 signaling (Andersen-Nissen et al., 2005; Johanesen and Dwinell, 2006). While the flagellin of *C. jejuni* does not play a significant role in the stimulation of cytokine production, an intact flagellar system is required for ERK kinase pathway activation. Watson and Galan (2005) reported that in the presence of chloramphenicol, an antibiotic that specifically blocks bacterial protein synthesis, ERK activation is diminished and IL-8 secretion is significantly reduced. Samuelson et al. (2013) reported that infection of INT 407 cells with a *C. jejuni flgBC* flagellar mutant, which is essential for the secretion of effectors from the flagellum, including CiaD, significantly reduces the amount of IL-8 in supernatants. These findings have been supported by studies in mice and pigs, where *C. jejuni* disease depends on the secretion of bacterial effectors from the flagellum (Neal-Mckinney and Konkel, 2012).

This study addresses the role of ERK1/2 in *C. jejuni* cell invasion and IL-8 secretion. Given that there is an overlap in the cellular components that comprise the *Campylobacter* invasion complex and the induction of the inflammatory response (e.g., β_1_ integrin → cSrc/FAK → paxillin → MAPK/ERK signaling), we hypothesized that inhibitors targeting key components would blunt both *C. jejuni* cell invasion and IL-8 secretion. While it is known that the nuclear translocation of phosphorylated ERK is required for IL-8 gene transcription, we further hypothesized that activated, cytoplasmic ERK is sufficient for actin reorganization and bacterial uptake. Determining the linkage between bacterial uptake and inflammatory cytokine production, and separating these two host processes, could clarify their significance in the onset of acute disease. Alternatively, determining whether active, phosphorylated cytoplasmic and/or nuclear ERK are necessary for bacterial uptake and inflammatory cytokine production provides a foundation for identifying the host cell proteins/genes involved in these processes. We initially investigated whether the CiaD effector is necessary for the nuclear translocation of ERK. We then used a phosphomimetic peptide that competitively inhibits the interaction of ERK1/2 with its nuclear transport receptor, Importin-7 (Imp7), thereby blocking ERK1/2 nuclear translocation, to test our hypotheses. Based on the results from this study, we propose that the MAPK/ERK signaling pathway is a potential therapeutic target for *C. jejuni* infections.

## 2 Materials and methods

### 2.1 Cell culture and reagents

The INT 407 (ATCC CCL-6) cell line was obtained from the American Type Culture Collection (ATCC). The cells were cultured in Minimal Essential Medium (MEM) supplemented with 10 mM sodium pyruvate, 20 mM glutamine, and 10% (v/v) fetal bovine serum (FBS). The cells were maintained at 37 °C in a humidified, 5% CO_2_ incubator.

### 2.2 Bacterial strains and growth conditions

*C. jejuni* strains used in this study are listed in Table 1. The *C. jejuni* wild-type (WT) strain 81-176 (a clinical strain recovered from a human patient), mutants, and complemented isolates were cultured on Mueller-Hinton agar plates with bovine blood in anticoagulant (5% citrate, Quad Five, Ryegate, MT, USA) (MH Blood) supplemented with the appropriate antibiotics (Δ*ciaD*, Δ*flgL*: Chloramphenicol 8 μg/mL, Δ*ciaD* + CiaD MKD-WT, Δ*ciaD* + CiaD-MKD-Ala-mutant, Δ*ciaD* + CiaD-MKD deletion, Δ*flgL* + FlgL WT: Hygromycin 250 μg/mL).

**Table 1:** *C. jejuni* strains used in this study.

Strains	Description	Reference
81-176	Wild-type, a diarrheagenic strain	(Samuelson et al., 2013)
Δ*ciaD*	*ciaD* deletion mutant	(Samuelson et al., 2013)
*ciaD*-comp	Δ*ciaD* mutant complemented with wild-type CiaD-FLAG	(Samuelson et al., 2013)
Δ*flgL*	*flgL* deletion mutant	(Samuelson et al., 2013)
*flgL*-comp	Δ*ciaD* mutant complemented with wild-type FlgL-FLAG	(Samuelson et al., 2013)
CiaD-MKD-Ala-mut	Δ*ciaD* mutant complemented with the alanine substitution in CiaD-MKD-3 × FLAG	This study
CiaD-MKD deletion	Δ*ciaD* mutant complemented with the MKD deletions in CiaD-3 × FLAG	This study
CiaD-MKD-WT	Δ*ciaD* mutant complemented with the wild-type MKD in CiaD-3 × FLAG	This study

### 2.3 Generation of the *C. jejuni* CiaD-MKD variant isolates

Three separate CiaD-MKD variant isolates were constructed for this study. We first created a CiaD-MKD WT construct in which *ciaD* is expressed by the constitutive pCysM promoter and contains a 3 × FLAG-tag at the C-terminal end. A 876 bp DNA fragment containing the pCysM-CiaD-3 × FLAG fragment was PCR amplified with primer pairs MEK4839/MEK4845 using a previous CiaD-complement plasmid as template (Samuelson et al., 2013) and the PCR product was cloned into the prRNA-HygR plasmid vector (Gourley et al., 2017) at the *Xba*I and BamHI sites, resulting in the plasmid prRNA-HygR-CiaD-MKD-WT-3 × FLAG. This new CiaD-complement plasmid (prRNA-HygR-CiaD-MKD-WT-3 × FLAG) was used as a template to make mutations in the MKD site (substitute K → A and L → A). Overlapping primers (MEK4841, MEK4842) containing the mutated MKD sites were used. Two separate PCR products were generated with primer pairs MEK4839/4842 and MEK4841/4845 and cloned into the prRNA-HygR vector at the *Xba*I and BamHI sites using the Infusion Snap Assembly Cloning Kit (Takar Bio Inc.). A gBlocks^TM^ gene fragment (IDT-DNA) containing the deletion of the MKD site was used to amplify the CiaD-MKD deletion fragment with primer pair MEK4839/4845 and cloned into prRNA-HygR at *Xba*I and BamHI sites. All new recombinant plasmid constructs were confirmed by plasmid sequencing (Plasmidsaurus Inc.). These three new CiaD-MKD variant plasmids were separately transformed into a *C. jejuni* Δ*ciaD* mutant, following the protocol mentioned in previous works (Samuelson et al., 2013). Briefly, plasmids were transformed into *C. jejuni* by electroporation and recovered in MH broth, followed by plating on MH Blood agar plates with antibiotic supplementation (Hygromycin, 250 μg/mL). Transformant colonies were selected randomly and confirmed by both PCR and sequencing of the isolates (Plasmidsaurus Inc.).

### 2.4 Bacterial motility

Bacterial biphasic cultures were prepared in Mueller-Hinton (MH) broth and grown on MH Blood agar plates overnight at 37 °C. The following day, cultures were collected, spun, and resuspended in 1 × PBS to obtain an optical density (OD) of 1. Allowing enough room for motility without restriction, 3 μL of each sample was spotted on an MH Soft agar plate (0.4% Bacto^TM^ Agar) without penetrating the agar. Plates were incubated for 24 h at 37 °C. The diameter of the motility zones was then measured (in mm) from one edge of the zone to the opposite edge.

### 2.5 Bacterial-cell invasion

The gentamicin-protection assay was performed following the methodology outlined in previous work (Konkel and Joens, 1989). Each well in a 24-well tissue culture tray (Costar^®^ 24-well Clear TC-treated Multiple Well Plate, Corning Inc.) was seeded with 4.5 × 10^5^ INT 407 cells. Before infection, cells were rinsed once with MEM-1% FBS. INT 407 cells were then inoculated with ~5 × 10^7^ CFU of a bacterial suspension in MEM-1% FBS [Multiplicity of Infection (MOI) = 100] and incubated for 3 h at 37 °C in humidified conditions (5% CO_2_). Following incubation, the infected cells were rinsed twice with 1 × PBS and incubated with MEM-1% FBS containing 250 μg/mL of gentamicin for 3 h to kill extracellular bacteria. The cells were rinsed with 1 × PBS and lysed with Triton X-100. A serial dilution was prepared for each culture and plated onto MH Blood agar plates in triplicate. The plates were incubated at 37 °C for 48 h, and the number of colonies for each dilution was recorded.

### 2.6 IL-8 secretion assay

INT 407 cells were infected with a bacterial suspension (MOI 10 or 100) and incubated in a humidified, 5% CO_2_ incubator at 37 °C. Depending on the assay, supernatant fluids were collected at 3 h (MOI 100) or following an overnight incubation of 16–18 h (MOI 10). The supernatants were centrifuged at 8,000 × *g* for 8 min to remove intact bacteria, followed by transferring to new tubes and frozen at −20 °C. Simultaneously, INT 407 cells were treated with phorbol myristate acetate and ionomycin (PMA/I), a cytokine stimulator that increases IL-8 production by cells, and used as a positive control. Uninfected cells were used as negative controls in the IL-8 assay.

IL-8 secretion assay was performed using an IL-8 ELISA kit following the manufacturer's guidelines (BD Biosciences Human IL-8 ELISA Set). Briefly, a 96-well assay plate (Corning^®^ 96-well Round Bottom Medium Binding Assay Plates, Corning Inc.) coated with capture antibody was diluted (1:1,000) in 1 × PBS (100 μL/well) and incubated overnight at 4 °C. The following day, wells were washed with the wash buffer (0.1% Tween-20 in 1 × PBS), blocked with 100 μL/well assay diluent (3% BSA in 1 × PBS), sealed, and incubated in the dark at room temperature (RT) for 1 h. A standard curve dilution was prepared using the human IL-8 stock standard provided with the ELISA kit. Assay samples and standard curve dilutions were aliquoted into a separate 96-well assay plate for efficient transfer and to prevent the primary assay plate from drying out between steps. After blocking, wells in the primary assay plate were washed three times, and 100 μL of samples and standard curve dilutions were added into each well, sealed, and incubated overnight at 4 °C with rocking. Following incubation, wells were washed five times, and 100 μL detection antibody + Sav-HRP reagent diluted in assay diluent (1:5,000) was added to each well, sealed, and incubated for 1 h in the dark at RT. After incubation, wells were washed seven times, 100 μL substrate solution [3,3′,5,5′-tetramethylbenzidine (TMB)] was added and incubated for 30 min in the dark at RT. Stop Solution (2.5 M H_2_SO_4_) was added (50 μL/well) after the incubation and mixed gently. At the final step, absorbance was read at 450 nm with a microplate reader (Bio-Tek Instruments, Inc. EL_x_808IU).

### 2.7 RNA extraction and RT-qPCR

Cell lysates were prepared from uninfected and *C. jejuni*-infected INT 407 cells at different time points (1, 3, and 6 h) post-infection. Cells were rinsed with sterile ice-cold 1 × PBS and transferred into microcentrifuge tubes, where 1 mL Trizol was added and kept in −80 °C freezer. Total RNA was extracted from Trizol containing cell lysates using Direct-zol RNA Miniprep Kit (Zymo Research) according to the manufacturer's protocol. Extracted RNA was treated with an additional DNase treatment using TURBO DNase^TM^ (Invitrogen, Thermo Fisher Scientific, Waltham, MA, USA) to remove any residual DNA from the extracted RNA samples. DNA-free RNA samples were quantified by NanoDrop, and 500 ng of each RNA sample was used to prepare cDNA using the iScript cDNA synthesis kit (Bio-Rad, Hercules, CA, USA). qPCR was performed with iTaq Universal SYBR Green Supermix (Bio-Rad), 500 nM of each primer, and 10 ng of cDNA on a CFX Opus 96 Real-Time PCR System (Bio-Rad). The primers used for RT-qPCR are listed in Table 2. The reaction condition was 95 °C for 30 s, followed by 40 cycles of 95 °C for 10 s, and 60 °C for 30 s. The 2^−ΔΔ*Ct*^ method was used to calculate the relative gene expression of *CXCL-8* compared to the housekeeping gene *GAPDH*.

**Table 2:** Primers used in this study.

Primer ID	Primer name	Sequence (5′-3′)^a, b^	Purpose
4839	rRNA-up-XbaI-pCysM-FW	TTGGATCACCTCCTTTCTAGAGTACTTTTTCAACTCAAGAAAG	CiaD-MKD amplification
4841	CiaD-MKD-Mut-FW	*CT*GATGAT*GC*TGAAAATAGG*GC*AAAC*GC*GACCATA	CiaD-MKD-Ala-mutant
4842	CiaD-MKD-Mut-RV	CCTATTTTCA*GC*ATCATC*AGCTGC*TATGCTATTT	CiaD-MKD-Ala-mutant
4845	rRNA-down-BamHI-3xFLAG-EcoRI-CiaD-RV	CAAGAGCTTTGGATCCTTATTTATCATCATCATCTTTATAATCAATATCATGATCTTTATAATCACCATCATGATCTTTATAATCGAATTCAAGCTTATCTTCGATATTTGC	CiaD-MKD-3XFLAG amplification
4823	CXCL8-FW	GAGAGTGATTGAGAGTGGACCAC	qPCR
4824	CXCL8-RV	CACAACCCTCTGCACCCAGTTT	qPCR
4837	GAPDH-FW	GTCTCCTCTGACTTCAACAGCG	qPCR
4838	GAPDH-RV	ACCACCCTGTTGCTGTAGCCAA	qPCR

^a^Restriction sites are underlined in the 4839 and 4845 primer sequences.

^b^The change in nucleotides compared to the wild-type CiaD MKD are italicized in 4841 and 4842 primer sequences.

### 2.8 ERK activation assays (ELISA and immunoblot)

INT 407 cells were inoculated with ~ 5 × 10^7^ CFU in 100 cm^2^ cell culture dish and incubated at 37 °C in a humidified, 5% CO_2_ incubator for 3 h. After the incubation, cell culture media was removed, washed with 1 × PBS, and 100 μL of 1 × cell lysis buffer mix was added. Cell lysis was done by shaking the cells in a shaker at 300 rpm for 10 min at RT. After the lysis, cell lysates were collected and used for ERK1/2 ELISA. The ERK1/2 ELISA was performed using the ERK1/ERK2 (Total/Phospho) InstantOne ELISA^TM^ Kit (Invitrogen, Thermo Fisher Scientific, Carlsbad, CA, USA) according to the user guidelines. Briefly, 50 μL of the sample lysates were added in triplicate to an InstantOne ELISA^TM^ microplate (provided with the ELISA kit), except two wells. One well was designated for 1 × cell lysis mix (negative control) and another well for the cell lysate provided with the ELISA kit (positive control). Next, 50 μL of prepared antibody cocktail was added to each of the wells of the microplate, sealed and shaken (300 rpm) for 1 h at RT. Following incubation, wells were washed with the wash buffer three times, 100 μL of detection reagent was added in each well and incubated on a shaker (300 rpm) for 15 min at RT. Finally, 100 μL of stop solution was added to each well, and absorbance was read with Bio-Tek Instruments, Inc. EL_x_808IU, set at 450 nm.

ERK activation was also determined by SDS-PAGE coupled with immunoblot analysis. The antibodies used were α-phosphorylated ERK1/2 (1:1000) (Phospho-p44/42 MAPK (T202/Y204), Rabbit, Cell Sig. Tech.), and α-actin (1:2,000) (Monoclonal anti-actin, anti-smooth muscle, Mouse, Sigma-Aldrich, St. Louis, MO, USA).

### 2.9 Paxillin immunoprecipitation

Dynabeads ^TM^ Co-Immunoprecipitation Kit (Invitrogen) was used to conjugate paxillin antibody according to the manufacturer's instructions. Briefly, 70 μL of paxillin antibody (05-417, 5H11, Sigma-Aldrich, St. Louis, MO, USA) was added to 10 mg of Dynabeads ^TM^ (magnetic beads) and incubated overnight at 37 °C with rotation. The antibody-coupled magnetic beads were stored at 4 °C until use. Cell lysates were prepared by passing the cells through a 21-gauge needle at least six times, followed by centrifuging at 2,600 rpm for 5 min at 4 °C. To prepare the antibody-coupled Dynabeads for immunoprecipitation (IP), 150 μL of beads were aliquoted into microcentrifuge tubes and washed with IP lysis buffer (25 mM Tris pH 7.4, 150 mM NaCl, 5% glycerol, 1% Triton-X-100). Eight hundred microliters of cell lysate was added to the antibody-coupled Dynabeads, gently mixed by inversion, and incubated overnight at 4 °C with rotation. After incubation, the tubes were placed on the magnetic stand (Invitrogen ^TM^ DynaMag ^TM^ −2 Magnet, Fisher Scientific), and the supernatant was collected and transferred to a new tube. Beads were washed for a total of five times with the wash buffer, mixed gently by inversion between the washes, and the supernatant was removed and discarded. Finally, 50 μL of ultrapure water and 50 μL of 2 × Laemmli buffer were added to each tube and mixed well. All samples were heated at 65 °C for 5 min, followed by placing the tubes in a magnetic stand. The supernatant (eluted samples) was collected and transferred to a new tube for SDS-PAGE and immunoblots.

### 2.10 SDS-PAGE, immunoblots, and densitometry

Samples were mixed 1:1 with 2 × Laemmli buffer and heated at 65 °C for 5 min. Heating at 65 °C was chosen to avoid detachment of phosphorylated groups. After heating, the samples were immediately placed on ice. Samples were run on a 12% gel via SDS-PAGE for 75 min at 150 V. Gels were then transferred to a membrane (1.5 h, 100 V) and prepared for Western blot analysis. Primary antibodies for phosphorylated protein were prepared in 3% bovine serum albumin (BSA). Primary antibodies for total protein were prepared in 5% non-fat dried milk (NFDM). Primary antibodies (Phospho-p44/42 MAPK (ERK1/2) (T202/Y204), Rabbit, Cell Sig. Tech. p44/42 MAPK (ERK1/2), Rabbit, Cell Sig. Tech., and α-paxillin antibody, clone 5H11, Mouse, Sigma-Aldrich) were diluted to 1:1,000. Ten milliliters of primary antibody was placed on the blots and incubated overnight at 4 °C with rocking. The following day, the blots were rinsed three times with 1 × TBST. The secondary antibodies (α-Rabbit IgG, α-Mouse IgG, Sigma-Aldrich) were diluted to 1:4,000 and incubated on the blots for 1.5 h at RT, with rocking. Blots were washed three times with 1 × TBST and once with diH_2_O before development (ECL™ Prime Western Blotting Detection Reagent, Amersham™ Cytiva, Marlborough, MA, USA). Blots were imaged with ImageQuant LAS 4000 mini Biomolecular Imager.

### 2.11 Confocal (immunofluorescence) microscopy

INT 407 cells were seeded at a density of 3.5 × 10 ^5^ cells/well on a 12 mm coverslip placed in each well of a 24-well tissue culture tray (Costar^®^ 24-well Clear TC-treated Multiple Well Plate, Corning Inc., Corning, NY, USA). Cells were transfected with a GFP-ERK1 plasmid (Plasmid #14747, Addgene, Watertown, MA, USA) using the Lipofectamine^®^ 3000 Transfection Kit as outlined by the manufacturer (Invitrogen). Four hours following the transfection, the medium containing the transfection reagent was replaced with 10% FBS-MEM and incubated overnight at 37 °C. The next day, cells were serum-starved for 4 h to quiet cellular activity, then infected with bacterial suspension (MOI 100) in MEM-1% FBS and incubated for 3 h at 37 °C in a 5% CO_2_ incubator. Following incubation, cells were washed with 1 × PBS, followed by fixing with 4% paraformaldehyde for 5 min at RT. The cells were quenched with 50 mM ammonium chloride for 5 min at RT, washed, and permeabilized with 1 mL permeabilization buffer (0.5% Triton-X-100 in 1 × PBS) for 15 min. After washing, a Mouse α-*C. jejuni* primary antibody diluted (1:500) in IF buffer (0.3% BSA, 0.1% Triton-X-100 in 1 × PBS) was added to each well and incubated for 45 min at RT, while rocking. The cells were washed, and a Goat α-Mouse (TRITC) secondary antibody diluted (1:500) in IF buffer was added to the wells and incubated for 30 min in the dark at RT, while rocking. After washing, the coverslips were removed from the tray, air dried, and mounted (cell-side down) on a glass slide with ProLong Diamond Antifade Mounting Media (Invitrogen, Thermo Fisher Scientific). Slides were stored overnight in the dark and then stored at 4 °C for future use. Images were taken within 2 days of staining.

For all analyses, the nuclear-to-cytoplasmic fluorescence ratio was calculated at the single-cell level as the total integrated density (IntDen) of the nucleus divided by the total IntDen of the cytoplasm (N/C ratio). Distributions of the N/C ratio were visualized using boxplots overlaid with jittered individual cell measurements. Plots were generated for the entire dataset (all experiments combined) and separately for each experiment (Expt. 1, Expt. 2, and Expt. 3). Statistical differences among groups were evaluated using a log-transformed response variable to improve normality and variance stability. For the combined dataset (all experiments), group effects were tested using a linear mixed-effects model of the form log(N/C ratio) ~ group + (1|Expt), where Expt (batch) was included as a random intercept to account for experiment-to-experiment variation. For each experiment analyzed separately, group effects were tested using a one-way analysis of variance model of the form log(N/C ratio) ~ group. Following each omnibus test, pairwise comparisons between all group means were performed using estimated marginal means with Tukey's HSD adjustment to control the family-wise error rate across all pairwise contrasts.

### 2.12 Other reagents and trypan blue viability assay

Inhibitor experiments were performed with samples treated with each inhibitor or the appropriate vehicle. The drugs (inhibitors) were added to the wells of a 24-well tissue culture tray (Costar^®^ 24-well Clear TC-treated Multiple Well Plate, Corning Inc.) 30 min before infection with *C. jejuni*. The inhibitors were used at the following concentrations: 50 μM PD98059 (MEK 1/2 inhibitor, Selleck, Houston, TX, USA), 10 μM TAE226 [Focal adhesion kinase (FAK) inhibitor, Selleck], 10 μg/mL PP2 (c-Src inhibitor, Sigma), 25 μM LY294002 [Phosphoinositide 3 (PI 3)-kinase inhibitor, Calbiochem, MilliporeSigma], 0.1 μM wortmannin (PI 3-kinase inhibitor, Enzo Biochem, Inc., Farmingdale, NY, USA), and 20 μM erlotinib (LC Laboratories, Woburn, MA, USA) (Hu et al., 2006; Neal-Mckinney and Konkel, 2012; Konkel et al., 2013; Eucker et al., 2014). Experiments were also performed with an EPE peptide, as it has been reported to block ERK1/2 nuclear translocation. The EPE peptide (GQLNHILGILGEPEQEDL) was conjugated in its N-terminal to myristic acid, and the C-terminal end was amidated (95% purity, Peptide 2.0 Inc., Chantilly, VA, USA). It was dissolved in DMSO (Sigma-Aldrich), and stored at −80 °C. The cells were rinsed and serum-starved in MEM without FBS for 3 h, and then the peptide (10, 20, and 30 μM) was added to the wells of a 24-well plate. The plates were incubated for 45 min to allow the peptide to penetrate the cells and then infected with *C. jejuni*. Supernatants were collected after a 3 h infection period and processed as outlined for the IL-8 secretion assay.

The trypan blue viability assay was performed in conjunction with the cell inhibitor and EPE peptide assays. Briefly, the supernatants were aspirated from the wells of the plate, and 100 μL of trypsin was added to each well. The plates were incubated for 5 min at RT, and then 400 μL of 1 × PBS was added to the well. A pipette was used to dislodge and suspend cells. A mixture of cells (10 μL) and 0.4% trypan blue (10 μL) was prepared, and cell viability was determined using an automated cell counter (EVE™, NanoEntek Inc., Waltham, MA, USA).

### 2.13 Statistical analysis

All experiments were performed at least three times. Mean and standard deviation (SD) were generated from each sample set. *P*-values were calculated using the Prism software (version 10.4.0) (GraphPad, La Jolla, CA, USA) using the appropriate statistical test for individual experiments indicated in the figure legends.

## 3 Results

### 3.1 *C. jejuni* triggers paxillin-associated ERK 1/2 activation

**Figure 1:**
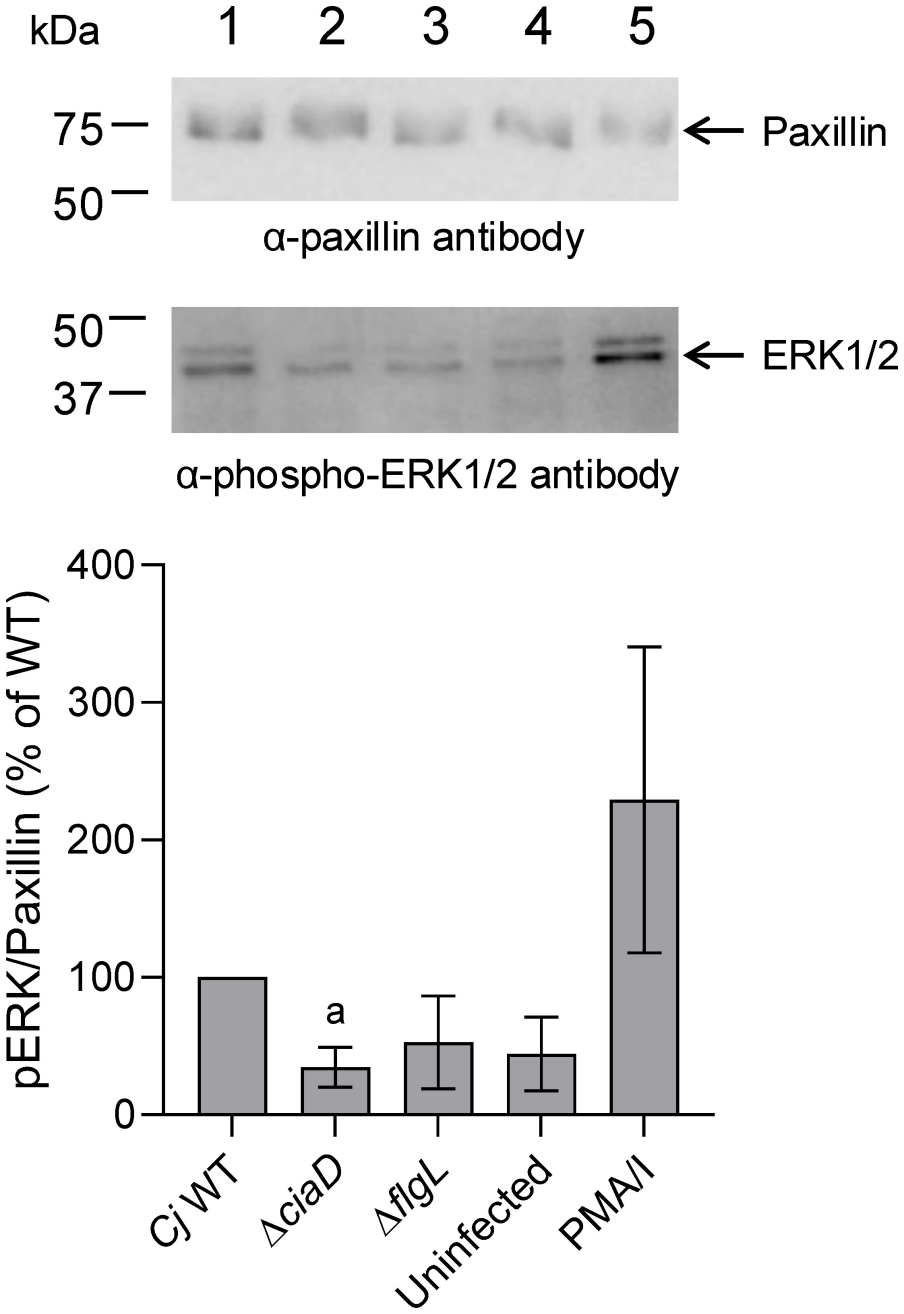
*C. jejuni* infection of INT 407 cells promotes pERK-paxillin association. Immunoprecipitation (IP) for paxillin was performed with the cell lysates collected from INT 407 cells infected with (1) *C. jejuni* wild-type (*Cj* WT), (2) Δ*ciaD* mutant, (3) Δ*flgL* mutant, (4) uninfected, and (5) uninfected + PMA/Ionomycin (PMA/I) after a 75-min infection period. Immunoblots for total paxillin and phosphorylated ERK1/2 (pERK 1/2) are shown for the IP samples. Densitometry was used to measure the intensity of the bands. The ratios between pERK and paxillin were calculated for each of the IP samples. The pERK/Paxillin ratio of the WT was normalized to 100%, and the ratios of other samples were adjusted to the WT. The mean ± SD for each sample (*n* = two replicates) is shown in a bar graph. A significant difference was noted between the WT and Δ*ciaD* infected sample (Unpaired *t*-test, ^a^*P* < 0.05).

ERK1/2 activation can occur via several mechanisms, including Protein Kinase C (PKC) signaling, a direct integrin-mediated pathway that bypasses paxillin, and a paxillin-dependent mechanism. Since *C. jejuni* binds to and invades cells at focal adhesions, where paxillin serves as a crucial scaffolding protein, we conducted immunoprecipitation (IP) followed by immunoblot assays to investigate whether paxillin is associated with phosphorylated ERK1/2 in response to *Campylobacter* infection. INT 407 cells were infected with the *C. jejuni* wild-type (WT) strain, Δ*ciaD* mutant, and Δ*flgL* mutant. Additional controls consisted of INT 407 cells treated with PMA/I to trigger IL-8 secretion and uninfected cells, respectively. IPs were performed with an α-paxillin antibody. SDS-polyacrylamide gel electrophoresis coupled with immunoblots was used to analyze phosphorylated ERK (pERK) to paxillin, and the ratio of pERK/paxillin was used to determine ERK1/2 association. A higher ratio of pERK/paxillin was observed with the INT 407 cells infected with the *C. jejuni* WT strain (100%), as well as with the PMA/I positive control (229.1 ± 111.3%), compared to uninfected cells (44.3 ± 26.9%) (Figure 1). No differences were noted in the pERK/paxillin ratio for the Δ*ciaD* (34.6 ± 14.6%) and Δ*flgL* mutants (52.7 ± 33.8%) compared to the uninfected cells (44.3 ± 26.9%). Based on these observations, we concluded that activated ERK1/2 is associated with paxillin upon *C. jejuni* infection.

### 3.2 *C. jejuni* induces ERK nuclear translocation and IL-8 secretion

**Figure 2:**
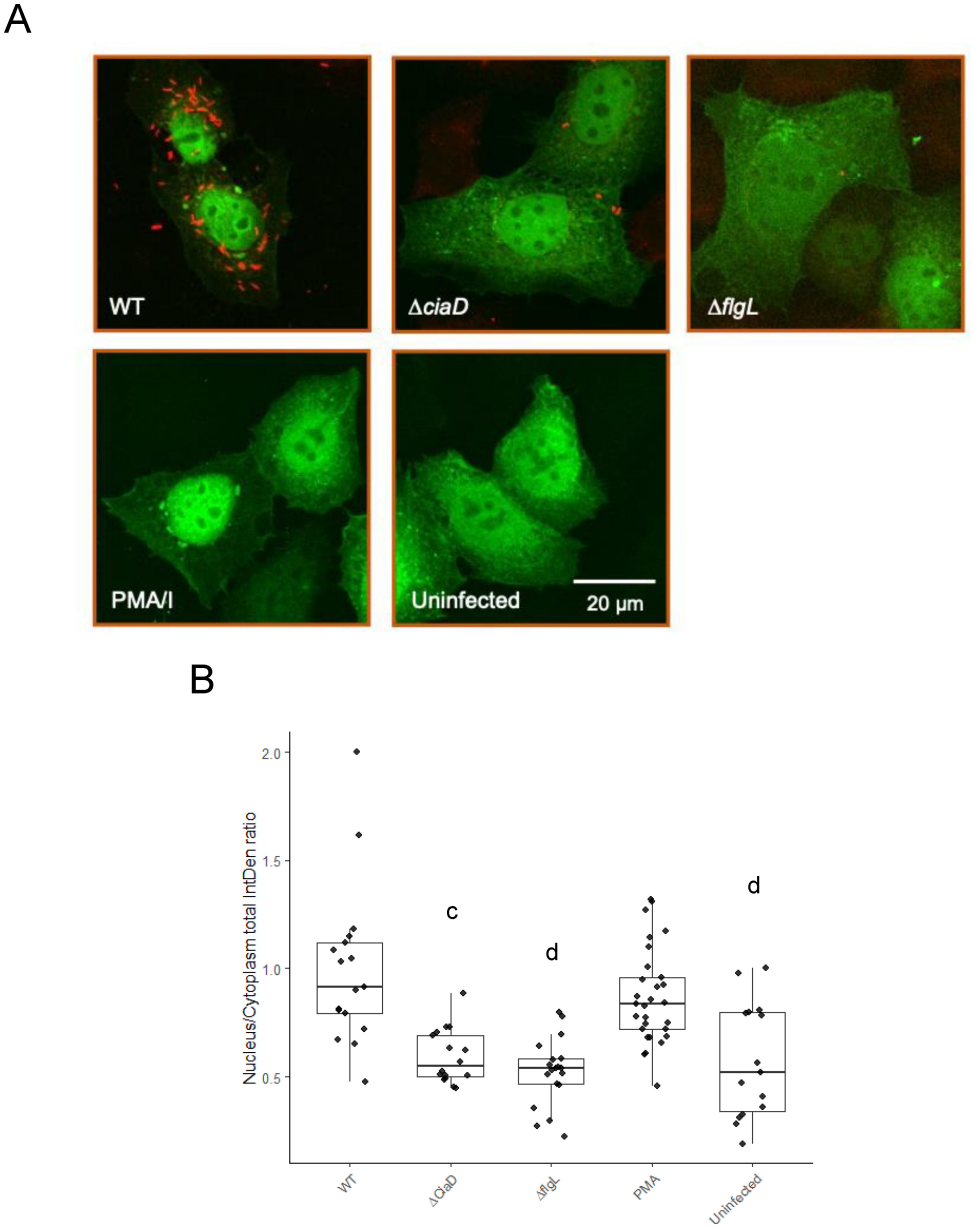
*C. jejuni* infection of INT 407 cells promotes ERK1/2 nuclear localization. INT 407 cells were transfected with GFP-ERK1 plasmid and infected with *C. jejuni* wild-type (WT), Δ*ciaD* mutant, Δ*flgL* mutant, uninfected + PMA/Ionomycin (PMA/I), and Uninfected for 3 h. *C. jejuni* were stained with a Mouse α-*C. jejuni* polyclonal antibody and a Goat α-Mouse (TRITC) secondary antibody. **(A)** Representative confocal microscopy images captured from the samples. Images were processed with Image J. **(B)** Distribution of the nuclear-to-cytoplasmic fluorescence ratio at the single-cell level [the total integrated density (IntDen) of the nucleus divided by the total IntDen of the cytoplasm (N/C ratio)]. Results are presented on a log scale, and each dot represents the N/C ratio of an individual cell. A significant decrease in ERK1/2 nuclear localization was observed with cells infected with Δ*ciaD* mutant, Δ*flgL* mutant, and uninfected cells compared to cells infected with the WT strain (Pair-wise group comparison with Tukey-HSD, ^c^*P* < 0.001, ^d^*P* < 0.0001).

Nuclear translocation of ERK is a critical event that alters gene expression, including the *CXCL-8* gene that encodes the inflammatory cytokine IL-8. Previously, we found that *C. jejuni* infection increased IL-8 secretion in both *in vitro* and *in vivo* models (Samuelson et al., 2013; Negretti et al., 2020). In this work, we determined the *CXCL-8* gene expression at different stages of infection (1 h, 3 h and 6 h post-infection). *CXCL-8* gene expression was detected at 3 h post-*C. jejuni* infection of INT 407 cells and further increased after 6 h of incubation (Supplementary Figure S1A). No increase was observed in *CXCL-8* gene expression for the cells inoculated with the *C. jejuni* Δ*ciaD* mutant or the Δ*flgL* mutant when compared to uninfected cells. We also measured the level of IL-8 secretion in INT 407 cells infected with *C. jejuni* isolates after 24 h of incubation. The level of secreted IL-8 from cells infected with the *C. jejuni* WT strain was significantly higher than cells infected with either Δ*ciaD* mutant and Δ*flgL* mutant isolates (Supplementary Figure S1B). Next, we wanted to determine if this *C. jejuni*-mediated *CXCL-8* gene expression, as well as IL-8 secretion, was associated with ERK nuclear translocation. For this, INT 407 cells were transfected with GFP-ERK1 plasmid and infected with *C. jejuni* WT strain, Δ*ciaD* mutant, and Δ*flgL* mutant. We then assessed the GFP-ERK1 localization by confocal microscopy. As expected, infection of the INT 407 cells with the *C. jejuni* WT strain significantly increased the level of ERK1 in the nucleus compared to cytosolic ERK1/2, whereas an increase in the level of nuclear ERK1 was not observed for the *C. jejuni* Δ*ciaD* and Δ*flgL* mutant-infected cells when compared to uninfected cells (Figure 2). These findings suggest that *C. jejuni* promotes ERK nuclear translocation that results in increased IL-8 secretion from the host cells. Based on the data in Figures 1, 2, we concluded that CiaD is responsible for both paxillin-associated ERK activation and nuclear translocation.

### 3.3 Alanine substitution at the MAP kinase docking site of CiaD decreases ERK 1/2 activation and nuclear translocation

ERK1/2 activation occurs through proteins containing a mitogen-activated protein kinase docking site (MAP kinase docking (MKD) or D site). We previously reported that the CiaD MKD site is important for *C. jejuni* cell invasion and for promoting IL-8 secretion from cells by infecting cells with a CiaD MKD deletion mutant (Samuelson et al., 2013). However, deletion of a stretch of residues can have a profound effect on a protein's conformation, ranging from instability to complete misfolding. Here, we generated a new CiaD variant harboring alanine substitutions of conserved residues to address whether the MKD site is required for ERK 1/2 activation and nuclear translocation. The reason for mutating the key residues in the MKD site to alanine is that it is a small, non-reactive amino acid that minimally affects a protein's secondary structure. The new *C. jejuni* CiaD variant (CiaD MKD alanine mutant) was found to contain the expected alanine substitution, as determined by PCR amplification of a DNA fragment using gene-specific primers, followed by sequencing of the amplified fragment (not shown). Immunoblot analysis using an α-FLAG antibody confirmed that the CiaD proteins in the CiaD MKD alanine mutant isolate (CiaD-MKD-Ala-mut), CiaD MKD deletion isolate (CiaD-ΔMKD), and complemented isolate (CiaD-MKD-WT) were synthesized in equal amounts in the *C. jejuni* transformants (Supplementary Figure S2). In parallel, the blots were probed with an α-CadF antibody to ensure even loading of the lysates. A soft agar motility assay confirmed that the *C. jejuni* WT, Δ*ciaD* mutant, and all CiaD-MKD variant (MKD-Ala-mut, ΔMKD, and MKD-WT) isolates were motile (Supplementary Figure S3). As predicted, the CiaD MKD Ala-mut isolate was deficient in host cell invasion and IL-8 secretion (Supplementary Figure S4). A Δ*flgL* mutant and *flgL* complement isolates were included in the invasion assay as negative and positive controls, respectively (Supplementary Figure S4A). The *flgL* gene encodes the hook-filament junction protein, which is essential for bacterial motility and the export of *Campylobacter* effector proteins (Cia proteins) from the flagellum ((Neal-Mckinney and Konkel, 2012)). Uninfected cells were included as a negative control for the IL-8 ELISA (Supplementary Figure S4B). In summary, these results confirmed that the CiaD MKD site is necessary for *C. jejuni* cell infection (host cell invasion and IL-8 secretion).

**Figure 3:**
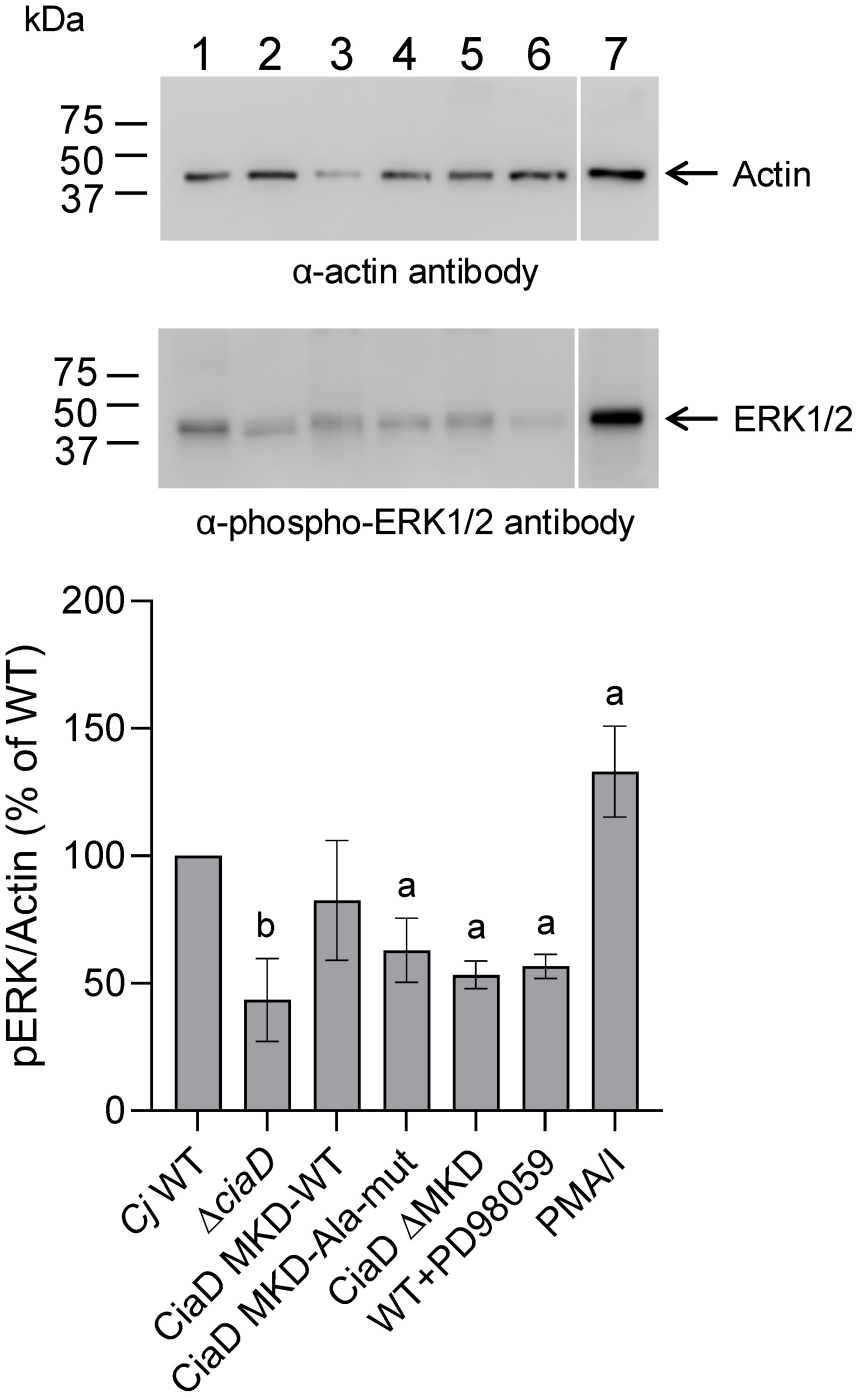
*C. jejuni* MKD mutants are impaired in ERK1/2 activation. INT 407 cells were infected with a (1) *C. jejuni* wild-type (*Cj* WT), (2) *ciaD* mutant (Δ*ciaD*), (3) CiaD complement isolate (CiaD MKD-WT), (4) CiaD MKD mutant (CiaD-MKD-Ala-mut), (5) CiaD MKD deletion mutant (CiaD ΔMKD), (6) WT infected + PD98059 treated (WT+PD98059), and (7) uninfected + PMA/Ionomycin (PMA/I). Cell lysates were collected from *C. jejuni-*infected cells after a 75-min infection period. A western blot was produced with the cell lysates, probing for actin and phosphorylated ERK1/2 (pERK 1/2), and densitometry was used to produce ratios between the bands. The pERK/actin ratio of the WT was normalized to 100%, and the ratios of other samples were adjusted to the WT. The plot shows the % of pERK/Actin ratios of cells infected with different *C. jejuni* isolates and PD98059-treated cells. The bars represent the mean ± SD for each sample (*n* = three replicates). Significant differences of pERK/actin ratios were observed for CiaD MKD variant samples compared to the WT (Unpaired *t*-test, ^a^*P* < 0.05; ^b^*P* < 0.01).

**Figure 4:**
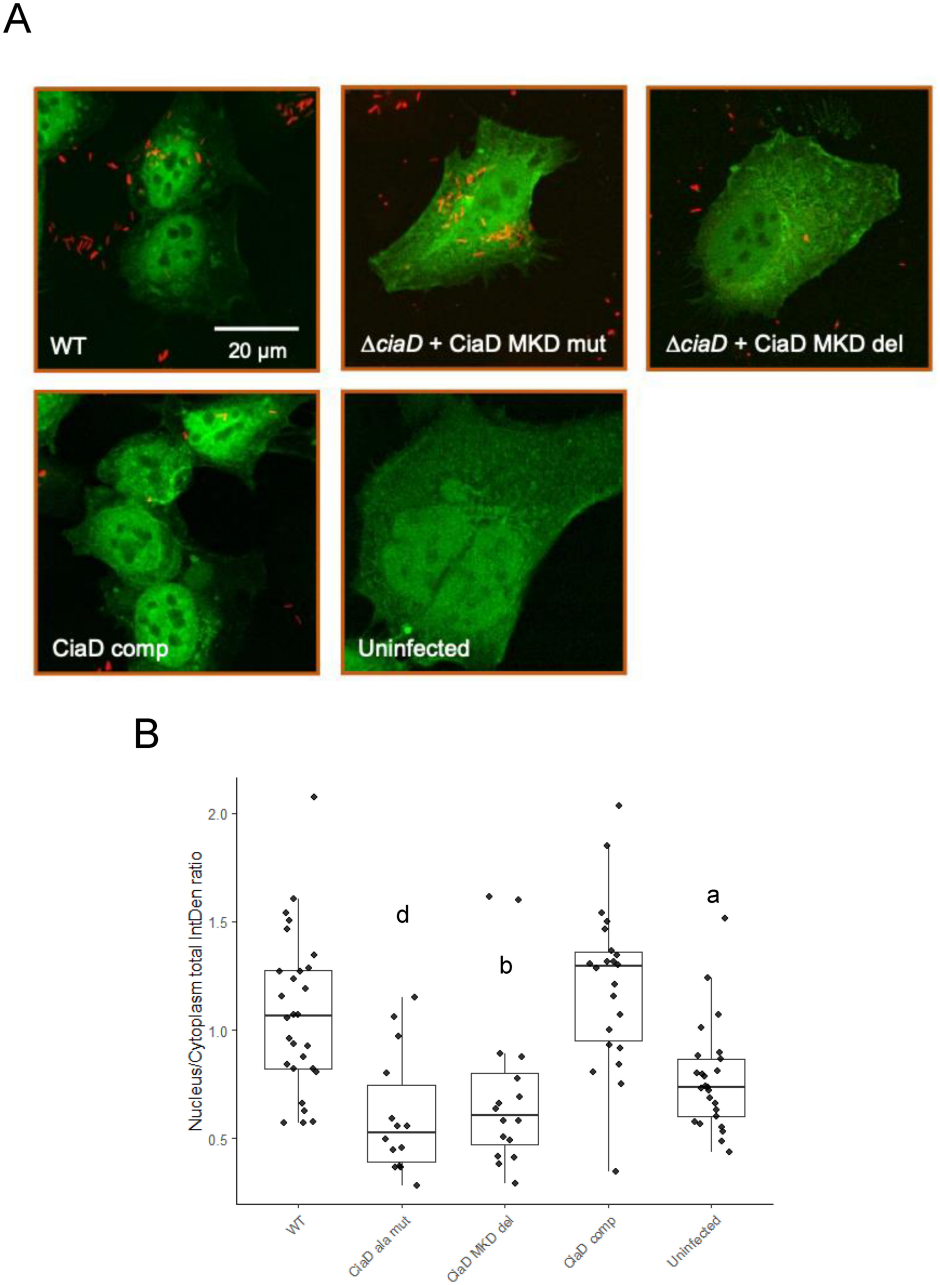
Alteration in the MAP kinase docking (MKD) of CiaD results in a decrease in ERK translocation to the nucleus. INT 407 cells were transfected with GFP-ERK1 plasmid and infected with various *C. jejuni* isolates for 3 h as outlined in “Section 2.” INT 407 cells were infected with the 1) *C. jejuni* wild-type (WT) strain, 2) CiaD MKD alanine mutant (CiaD MKD mut), 3) CiaD MKD deletion mutant (CiaD MKD del), and 4) CiaD complement isolate (CiaD comp). Uninfected cells served as a negative control. *C. jejuni* were stained with a Mouse α-*C. jejuni* polyclonal antibody and a Goat α-Mouse (TRITC) secondary antibody. **(A)** Representative confocal microscopy images captured from the samples. Images were processed with Image J. **(B)** Distribution of the nuclear-to-cytoplasmic fluorescence ratio at the single-cell level [the total integrated density (IntDen) of the nucleus divided by the total IntDen of the cytoplasm (N/C ratio)]. Results are presented on a log scale. Each dot represents the N/C ratio of an individual cell. A significant decrease in ERK1/2 nuclear localization was observed with cells infected with CiaD-MKD-Ala-mut, CiaD MKD deletion mutant, and uninfected cells compared to cells infected with the *C. jejuni* wild type (WT) strain (Pair-wise group comparison with Tukey-HSD, ^a^*P* < 0.05; ^d^*P* < 0.0001).

To determine if the CiaD MKD Ala-mut is necessary for ERK1/2 activation, INT 407 cells were infected with the various *C. jejuni* isolates and immunoblot analysis was performed with probing for actin and phosphorylated ERK1/2 (pERK1/2). Actin was used as a loading control. PD98059 is a non-ATP competitive MEK inhibitor that blocks ERK1/2 activation and was used as a negative control. PMA/I promotes ERK1/2 activation and was used as a positive control. In contrast to the infection of INT 407 cells with a *C. jejuni* WT strain (100%), infection of INT 407 cells with the CiaD MKD Ala-mut did significantly decrease the level of activated (phosphorylated) ERK1/2 in cells (62.9 ± 12.6%) (Figure 3). Moreover, an increase in phospho-ERK1/2 was also observed in cells infected with the CiaD-complemented isolate (CiaD-MKD-WT) (82.6 ± 23.5%) and cells treated with PMA/I (133.1 ± 17.9%). In a separate experiment, a significant increase was observed in activated ERK1/2 in INT 407 cells infected with the WT strain and CiaD-complemented isolate compared to uninfected cells, as judged by an ERK1/ERK2 ELISA (Supplementary Figure S5). In contrast, no differences were noted in levels of activated ERK1/2 in INT 407 cells infected with either the Δ*ciaD* or Δ*flgL* deletion mutants compared to uninfected cells (Supplementary Figure S5). To determine if the CiaD MKD Ala-mut facilitates some ERK1/2 nuclear translocation, INT 407 cells were infected with various *C. jejuni* isolates. Uninfected cells served as a negative control. In contrast to the infection of INT 407 cells with a *C. jejuni* WT strain, for which an increase in ERK1/2 was observed in the nucleus, no difference was observed in the level of nuclear ERK1/2 for the CiaD MKD Ala-mut infected cells and uninfected cells (Figure 4).

### 3.4 Treatment of cells with the EPE peptide reduces *C. jejuni* cell invasion and lessens host cell IL-8 secretion

**Figure 5:**
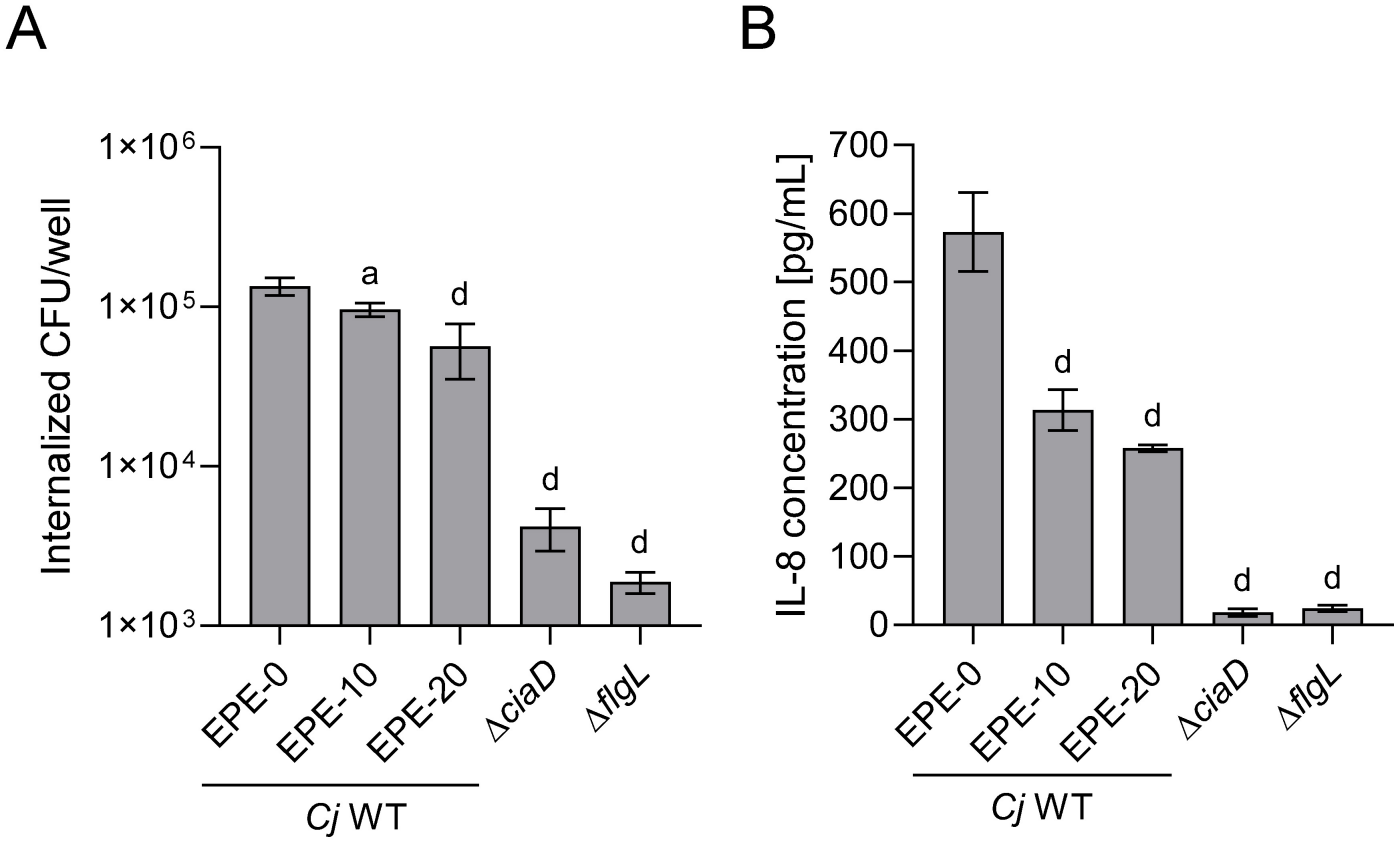
The EPE peptide reduces *C. jejuni* cell invasion and IL-8 secretion. INT 407 cells were treated with different concentrations of EPE peptide (0, 10, and 20 μM) for 45 min, followed by infection with *C. jejuni* strains: wild-type (*Cj* WT), Δ*ciaD* mutant, and Δ*flgL* mutant. *C. jejuni* internalization and IL-8 secretion were measured by the gentamicin-protection assay and ELISA, respectively. **(A)** The number of internalized bacteria in EPE peptide-treated and untreated cells. There was a significant reduction in *C. jejuni* internalization when cells were treated with EPE peptide (EPE-10 and EPE-20) compared to no treatment (EPE-0) (one-way ANOVA followed by Dunnett's multiple comparison test, ^c^*P* < 0.001; ^d^*P* < 0.0001). **(B)** The amount of IL-8 in supernatants collected from *C. jejuni*-infected and EPE peptide-treated and untreated cells at 6 h post-infection. A significant reduction in IL-8 secretion was observed for cells treated with EPE-peptide (EPE-10 and EPE-20) compared to untreated cells (EPE-0) (one-way ANOVA followed by *post-hoc* Dunnett's analysis, ^c^*P* < 0.001; ^d^*P* < 0.0001). The gray bars indicate the mean ± SD for each sample (*n*= four replicates).

**Figure 6:**
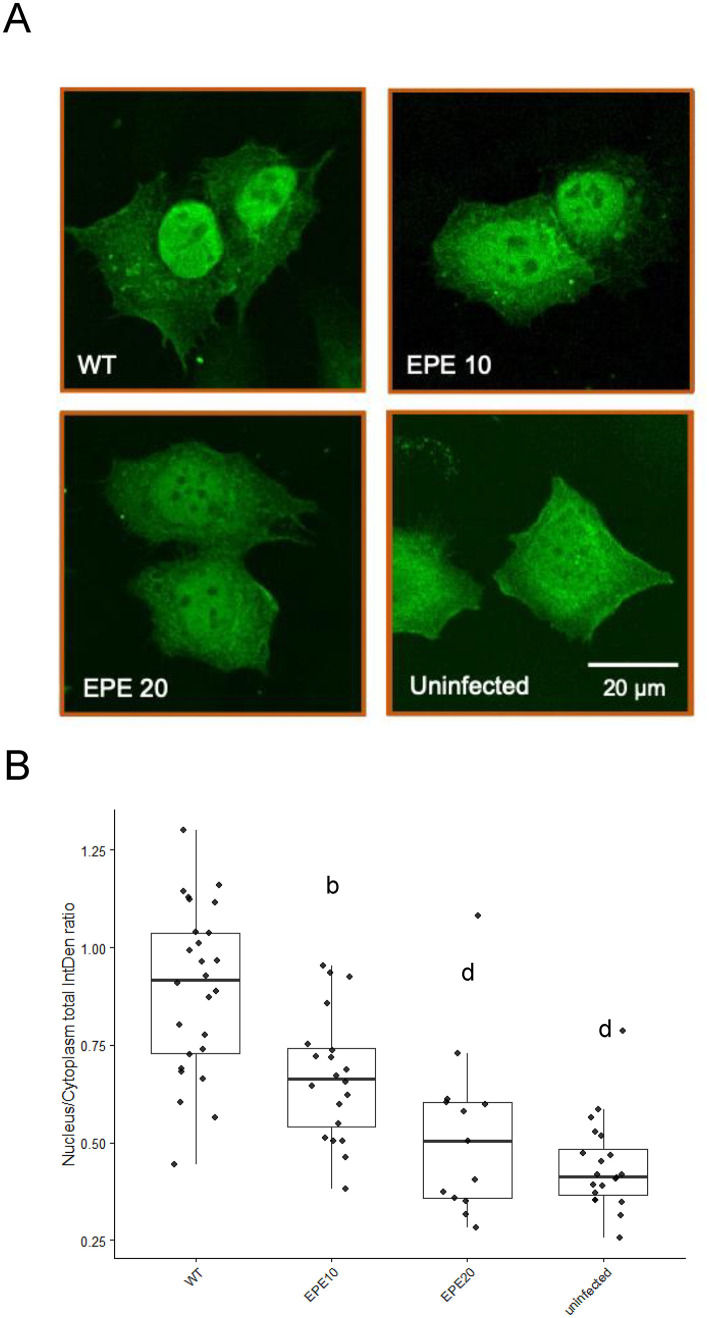
EPE peptide reduces ERK1/2 nuclear localization. INT 407 cells were transfected with GFP-ERK1 plasmid and infected with the *C. jejuni* wild-type strain (*Cj* WT) with no EPE peptide (top panel, upper left image) and the *Cj* WT strain with 20 μM (EPE 20) and 30 μM (EPE 20) of the EPE peptide. Uninfected cells served as a negative control. **(A)** Representative confocal microscopy images captured from the samples. Images were processed with Image J. **(B)** Distribution of the nuclear-to-cytoplasmic fluorescence ratio at the single-cell level [the total integrated density (IntDen) of the nucleus divided by the total IntDen of the cytoplasm (N/C ratio)]. Results are presented on a log scale. Each dot represents the N/C ratio of an individual cell. A significant decrease in ERK1/2 nuclear localization was observed with infected cells treated with EPE 10, EPE 20 and uninfected cells compared to infected cells without treatment (Pair-wise group comparison with Tukey-HSD, ^b^*P* < 0.01; ^d^*P* < 0.0001).

The classical mechanism of protein translocation into the nucleus involves nuclear localization signal (NLS)-mediated binding to importin-α and importin-β. However, ERK1/2 lacks a canonical NLS. The translocation of ERK1/2 to the nucleus involves the phosphorylation of two serine residues within a nine amino acid sequence (SPS motif, Ser-Pro-Ser), termed nuclear translocation signal (NTS). The phosphorylation of ERK1/2's SPS motif allows it to bind to Imp7, which escorts the kinases into the nucleus. Previous work has shown that the myristoylated, NTS-derived phosphomimetic EPE (Glu-Pro-Glu) peptide inhibits the interaction between Imp7 and ERK1/2 (it competitively inhibits the interaction of ERK1/2 with Imp7), thereby preventing ERK1/2 nuclear translocation (Plotnikov et al., 2015; Birkner et al., 2017; Flores et al., 2019). Additionally, reports have shown that ERK1/2 nuclear translocation is crucial for inducing IL-8 synthesis and secretion by activating transcription factors (Liu et al., 2000; Jijon et al., 2002; Lee et al., 2006; Wortzel and Seger, 2011). To address whether blocking the transport of phosphorylated ERK1/2 to the nucleus affects *C. jejuni* cell invasion and/or IL-8 secretion, assays were performed with the EPE peptide. Specifically, INT 407 cells were incubated with 10 and 20 μM of the EPE peptide to block ERK1/2 nuclear translocation. Negative controls consisted of a *C. jejuni* Δ*flgL* flagellar hook mutant and uninfected cells. A significant decrease in *C. jejuni* cell invasion (10 μM EPE, ~28% reduction; 20 μM EPE, ~58% reduction) and host cell IL-8 secretion (10 μM EPE, ~45% reduction; 20 μM EPE, 55% reduction) was observed in the presence of the EPE peptide compared to *C. jejuni*-infected cells in the absence of the peptide (Figure 5). These observations were supported by the observation of a decrease in nuclear ERK in *C. jejuni*-infected cells in the presence of the EPE peptide compared to *C. jejuni*-infected cells in the absence of the peptide (Figure 6). Taken together, these data indicate that the EPE peptide inhibits nuclear translocation of ERK1/2, and that ERK1/2 transport to the nucleus is required for *C. jejuni* cell invasion and the induction of host cell IL-8 secretion. Additionally, these findings indicate that both cytoplasmic and nuclear ERK are required for maximal *C. jejuni* cell invasion.

### 3.5 Drug and peptide inhibitors targeting ERK activation and nuclear translocation lessen host cell IL-8 secretion

**Figure 7:**
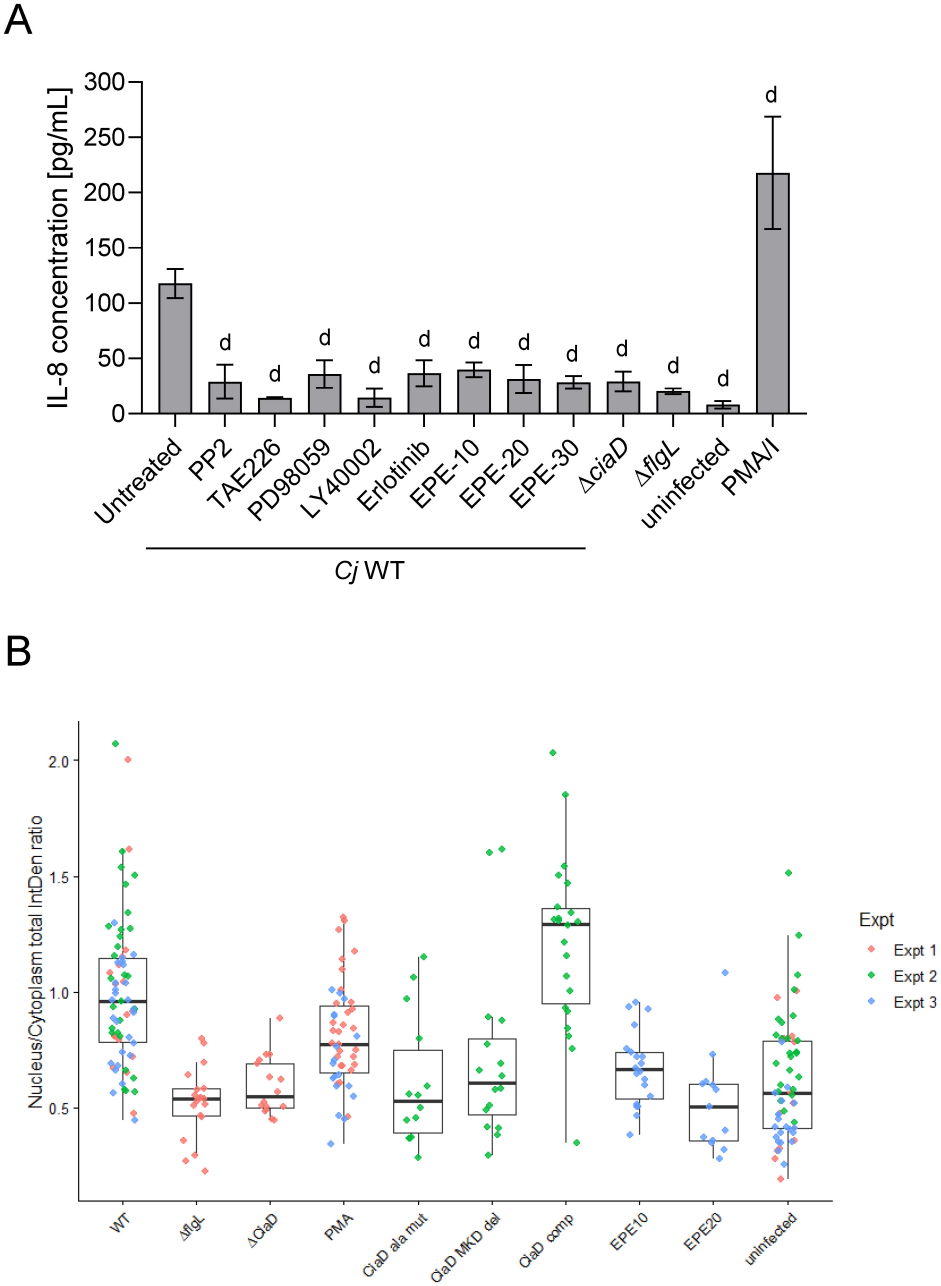
Drugs directed against host proteins associated with focal adhesion function and/or the MAPK/ERK signaling pathway inhibit *C. jejuni*-induced production of IL-8. **(A)** INT 407 cells were infected with the *C. jejuni* wild-type strain in the presence of drugs that target cellular proteins [PP2 (c-Src inhibitor), TAE 226 (FAK inhibitor), erlotinib (EGF receptor kinase inhibitor), LY294002 (PI 3-kinase inhibitor), wortmannin (PI 3-kinase inhibitor), and PD98059 (MEK1 activation inhibitor)], and the EPE peptide at different concentrations (10, 20 and 30 uM) that inhibits IMP7 and ERK1/2 interaction and blocks phosphorylated ERK1/2 nuclear translocation. Uninfected cells served as a negative control. Supernatants were collected at 3 h post-infection, and IL-8 was measured via ELISA. Results are presented as the amount of IL-8 detected in the supernatants (pg/mL). **(B)** Composite figure from three experiments showing relative levels of ERK1/2 nuclear localization in *C. jejuni*-infected and uninfected INT 407 cells. Experiment #1) *C. jejuni* wild-type (WT) infected cells, Δ*flgL* mutant infected cells, Δ*ciaD* mutant infected cells, PMA/IO-treated cells, and uninfected cells; Experiment #2) WT infected cells, CiaD MKD Ala-mut infected cells, CiaD MKD del (ΔMKD) mutant infected cells, CiaD complemented infected cells, and uninfected cells; and Experiment #3) WT infected cells, WT infected cells treated with the EPE peptide (EPE 20), and uninfected cells. The figure shows the boxplots of the various samples (WT = Distribution of the nuclear-to-cytoplasmic fluorescence ratio at the single-cell level) [the total integrated density (IntDen) of the nucleus divided by the total IntDen of the cytoplasm (N/C ratio)]. Cells were treated with phorbol myristate acetate and ionomycin (PMA/IO), which increases IL-8 production by cells, as a positive control. Each dot represents the N/C ratio of an individual cell.

Targeting components of the focal adhesion and ERK1/2 signaling pathway with inhibitors is being explored as a potential therapeutic strategy for inflammatory diseases and certain cancers (Martin-Vega and Cobb, 2023). We used different drugs targeting focal adhesions and the EPE peptide in an assay to compare the effectiveness of these molecules in reducing IL-8 secretion from *C. jejuni*-infected cells. The inhibitors included PP2 (c-Src inhibitor), TAE226 (FAK inhibitor), erlotinib (Epidermal growth factor (EGF) receptor kinase inhibitor), LY294002 (PI3-kinase inhibitor), wortmannin (PI 3-kinase inhibitor), and PD98059 (MEK1 activation inhibitor), as well as the EPE peptide (blocks nuclear translocation of phosphorylated ERK1/2). The assay was designed to minimize the potential toxic effects of inhibitors on the cells; INT 407 cells were pretreated with each potential inhibitor for 45 min, infected with *C. jejuni*, and the supernatants were collected after an additional 3 h incubation. The amount of IL-8 in the supernatants was determined by ELISA, and the trypan blue exclusion assay was used to assess cell viability. A decrease in IL-8 was observed in the *C. jejuni*-infected cells pre-treated with the PP2, TAE226, erlotinib, LY294002, wortmannin, and PD98059 inhibitors, confirming the involvement of the focal adhesion components in *C. jejuni*-induced IL-8 production (Figure 7A). Moreover, a reduction in IL-8 was again observed with the supernatant from *C. jejuni*-infected cells in the presence of the EPE peptide compared to *C. jejuni*-infected cells in the absence of the peptide. The reduction in IL-8 levels upon treatment of the cells correlated with a reduction in ERK1/2 nuclear translocation (Figure 7B). However, treatment of the cells with the EPE peptide did not reduce the level of nuclear ERK to the level observed for cells infected with the *C. jejuni* Δ*ciaD* mutant, which may partially explain why a greater reduction in *C. jejuni* cell invasion was not observed in the presence of the peptide. None of the inhibitors resulted in detectable changes in host cell viability compared with untreated cells (Supplementary Figure S6). This finding confirmed that any drug or peptide that inhibits ERK1/2 translocation will blunt *C. jejuni*-mediated IL-8 secretion from the host cells and is also likely to reduce *C. jejuni* cell uptake.

## 4 Discussion

**Figure 8:**
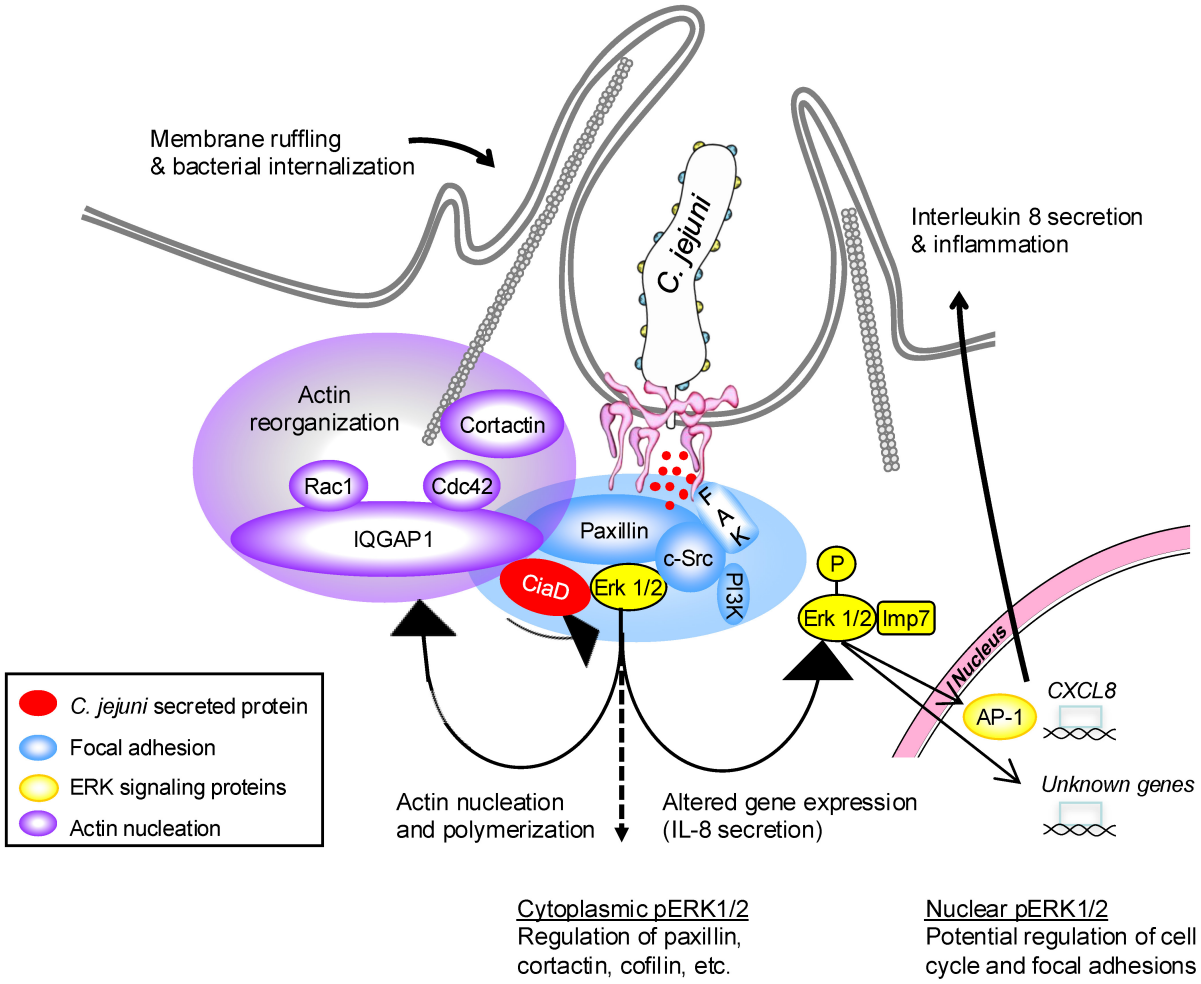
A model of the *Campylobacter* invasion complex involved in bacterial uptake and IL-8 secretion from epithelial cells. The model is based on published data, results from this project, and cellular components that are proposed to regulate *C. jejuni* cell invasion and IL-8 secretion. In this study, we found that inhibitors of FAK (TAE 226), c-Src (PP2), and ERK1/2 (PD98059) result in a reduction in IL-8 gene transcription and a decrease in the level of IL-8 secreted from *C. jejuni*-infected host cells. Previous work has shown that CiaD binds to the host cell protein IQGAP1 (a Ras GTPase-activating-like protein), stimulating actin reorganization and *C. jejuni* internalization. The activation of ERK1/2, which also binds to IQGAP1, occurs through proteins containing a MAP kinase docking (MKD) site. We show that an MKD site in the *C. jejuni* effector CiaD is required for *C. jejuni* cell invasion and IL-8 secretion from INT 407 cells. We propose that activated (phosphorylated) ERK1/2 in the cytosol activates key components required for *C. jejuni* invasion, including paxillin, cortactin, and cofilin (dotted arrow), and that ERK1/2 nuclear translocation is required for IL-8 secretion. Based on the findings in this study, we conclude that activated ERK1/2 in the cytosol directly modulates the activity of host cell proteins that contribute to *C. jejuni* cell invasion and that nuclear ERK1/2 acts as a critical signaling mediator that promotes IL-8/CXCL8 transcription and subsequent secretion, as well as regulates the cell cycle, resulting in focal adhesion and cytoskeletal remodeling and contributing to *C. jejuni*-mediated uptake.

The primary focus of this study was to investigate whether ERK1/2 activation occurs through a paxillin-dependent mechanism and to determine if inhibiting the nuclear translocation of activated ERK1/2 affects the ability of *C. jejuni* to invade epithelial cells. Our findings indicate that paxillin is a key scaffolding protein that recruits ERK1/2. More specifically, we observed an increased level of phosphorylated ERK (pERK1/2) associated with paxillin in INT 407 cells infected with the *C. jejuni* WT strain vs. cells infected with the Δ*ciaD* mutant, as judged by immunoprecipitation (IP) of paxillin, followed by immunoblotting with antibodies reactive against phospho-ERK and paxillin (Figure 1). Further, *C. jejuni* cell infection and CiaD effector delivery to the cytosol of host cells led to the nuclear transport of phosphorylated ERK1/2 (Figures 2–4). We also found that the treatment of cells with the EPE peptide, which competitively inhibits the interaction of ERK1/2 with Imp7 and blocks phosphorylated ERK1/2 nuclear translocation, reduces *C. jejuni* cell invasion and lessens host cell IL-8 secretion. Together, these and previous findings suggest that *C. jejuni* uses its effector molecules (Cia proteins, e.g., CiaD) to alter ERK signaling (ERK1/2 activation) through focal adhesion components (paxillin, central scaffold protein), and ERK nuclear translocation is necessary for host cell IL-8 secretion (Figure 8). Additionally, we discovered that both cytosolic and nuclear ERK1/2 is required for maximal *C. jejuni* cell invasion (Figure 8). Both the Activator Protein-1 (AP-1) and NF-κB transcription activators are required for IL-8 gene transcription. While various factors can activate the AP-1 transcription factor, including oxidative stress and infection, the NF-κB transcription factor is known to be activated by several *C. jejuni* factors, including JlpA, CdtABC, and peptidoglycan (Hickey et al., 2000; Jin et al., 2003; Zilbauer et al., 2007). Our data support the proposal that the ERK activates AP-1.

In general, cytoplasmic ERK1/2 is associated with altering the function of cytoskeletal components and scaffold proteins, whereas nuclear accumulation of ERK1/2 is important for cell proliferation (Birkner et al., 2017). We predicted that activated, cytosolic ERK1/2 would be required for maximal *C. jejuni* invasion of cells, as it phosphorylates paxillin and cortactin (Yoon and Seger, 2006). Paxillin and cortactin are known to be part of the *Campylobacter* invasion complex (Monteville et al., 2003; Samuelson and Konkel, 2013; Klappenbach et al., 2021). We have previously shown that ERK1/2 activation is responsible for the phosphorylation of cortactin at serine residues S405 and S418 (Samuelson and Konkel, 2013). Cortactin has also been shown to be necessary for the maximal *C. jejuni* invasion of host cells using siRNA to cortactin and cortactin-phosphorylation null constructs (S405A, S418A, S405/418A) coupled with the gentamicin protection assay (Samuelson and Konkel, 2013). Serine phosphorylation of cortactin is recognized to lead to the recruitment of N-WASP, activation of Arp2/3, and actin remodeling (Cosen-Binker and Kapus, 2006).

Although paxillin and cortactin are known components of the *Campylobacter* invasion complex, we propose that activated, cytosolic ERK1/2 regulates other cellular proteins that have yet to be recognized for their role in *C. jejuni* cellular uptake, most notably, cofilin. Actin-filament dynamics in cells are known to be modulated by cofilin, and the regulation of cofilin activity has been shown to contribute to both viral and bacterial uptake through the formation of filopodia and lamellipodia (Zheng et al., 2016). In the case of Herpes Simplex Virus 1 (HSV-1) entry into cells, the activation of ERK1/2 in response to HSV-1 infection leads to cofilin phosphorylation and virus-induced filopodium formation (Zheng et al., 2014). Active ERK1/2 with F-actin was observed at the base of the filopodia. Notably, in this study, ERK1/2 activation and cofilin phosphorylation were inhibited in HSV-1-infected cells treated with LY294002 (PI 3-kinase inhibitor) and PP2 (Src kinase inhibitor). Bacterial pathogens that utilize a zipper (*Listeria* and *Yersinia* spp.) or trigger mechanism (e.g., *Shigella, Salmonella*) also exploit cofilin activity (Cossart and Sansonetti, 2004; Zheng et al., 2016). In the case of *Listeria monocytogenes*, the process of phagocytosis requires both the activation and deactivation of cofilin, involving the interaction of InlB with the host cell Met receptor, followed by the activation of host cell phospholipase D (Han et al., 2011; Zheng et al., 2016). In summary, pathogens alter the activity of cofilin in the process of cell invasion to alter actin dynamics and formation of the phagocytic cup—too little active cofilin results in excessive F-actin polymerization, and too much active cofilin causes a loss of F-actin disassembly. Based on the published literature, studies are warranted to investigate the role of cofilin in *C. jejuni* in the formation of the actin-rich protrusion and resolution of the endocytic cup.

We found that inhibiting the nuclear translocation of phospho-ERK1/2 with the phosphomimetic EPE peptide, which blocks the interaction of Imp7 with ERK1/2, reduces both *C. jejuni* cell invasion and IL-8 secretion. It was expected that inhibiting ERK1/2 nuclear translocation would decrease IL-8 levels in supernatants collected from *C. jejuni*-infected cells; nuclear localization of ERK1/2 is required for IL-8 synthesis and secretion because it activates nuclear factors that induce IL-8 gene transcription. However, it was unexpected that blocking activated ERK1/2 from entering the nucleus would decrease *C. jejuni* cell invasion. One possible explanation for why blocking ERK nuclear translocation reduces *C. jejuni* invasion relates to the cell cycle phase. Nuclear ERK1/2 is crucial for controlling cell cycle progression; it persists during the entire G_1_ phase, and levels must be sustained to drive the G_1_/S-phase transition (Mebratu and Tesfaigzi, 2009). Relevant is that *C. jejuni* targets epithelial cells in the G_1_ phase of the cell cycle (Talukdar et al., 2025). Specifically, *C. jejuni* infection of INT 407 cells alters the rate of cell cycle progression and the distribution of focal adhesions, resulting in a greater number of cells in the G_1_ cell cycle phase, with focal adhesions located in the cell periphery. An *in vivo* study has revealed that the cells at the tip of the intestinal villi are in the G_1_ phase of the cell cycle (Moor et al., 2018), underscoring that this pathogen has evolved to manipulate host cell structure and function for its benefit. Given that inhibiting the nuclear translocation of phospho-ERK1/2 reduces *C. jejuni* cell invasion, we performed an experiment to determine whether activating ERK1/2 with PMA/I would increase the invasion of the wild-type isolate. We did not observe a statistical difference in cell invasion between untreated and PMA/I-treated samples (data not shown). While kinetic assays could reveal differences in *C. jejuni* invasion of untreated and PMA/I-treated cells, it is also possible that the *C. jejuni* wild-type rapidly activates ERK1/2 to a level more than that needed for maximal cell invasion. Regardless, additional studies are required to identify the genes encoding the components of the *Campylobacter* invasion complex and/or those regulating the processes involved in *C. jejuni* cell invasion. One experiment that would shed light on the cellular genes encoding products involved in *C. jejuni* invasion would be to perform bulk RNA sequencing of *C. jejuni*-infected cells treated with the EPE peptide.

Multiple drugs were used to target host cell proteins involved in focal adhesion function and/or the MAP (Ras/Raf/MEK/ERK) signaling pathway to inhibit *C. jejuni*-driven cellular signaling processes, particularly IL-8 synthesis and secretion. The inhibitors used in this study included PP2 (c-Src inhibitor), TAE226 (FAK inhibitor), erlotinib (EGF receptor kinase inhibitor), LY294002 (PI 3-kinase inhibitor), wortmannin (PI 3-kinase inhibitor), and PD98059 (MEK1 activation inhibitor), and the EPE peptide (blocks phosphorylated ERK1/2 nuclear translocation). Notably, FAK-Src phosphorylation of paxillin can lead to the recruitment of MAP kinase ERK1/2 (Ishibe et al., 2003). LY294002 and wortmannin were included in the assay because focal adhesions play a crucial role in recruiting and activating PI 3-kinase (Wang et al., 2024), and the PI 3-kinase pathway activates Akt and NF-κB, leading to increased IL-8 production (Mao et al., 2025). Erlotinib, an inhibitor of the EGF receptor, was used because previous works have shown that the activation of focal adhesion recruits the EGF receptor (Giancotti and Ruoslahti, 1999; Zouq et al., 2009), and because *C. jejuni* infection of cells results in EGF receptor phosphorylation (Konkel et al., 2013). Throughout these experiments, kinase activity and ERK nuclear translocation inhibitors were used at concentrations reported in publications (Hu et al., 2006; Konkel et al., 2013; Eucker et al., 2014). None of the inhibitors affected cell viability as judged by the trypan blue exclusion assay. The fact that the drugs targeting focal adhesions used in this study reduced IL-8 synthesis/secretion, and that these inhibitors also reduced *C. jejuni* cell invasion (Supplementary Figure S7), illustrates the significance of ERK activation in establishing an infection. The intense inflammatory response surely contributes to intestinal barrier disruption, permitting *C. jejuni* access to the basolateral surface of cells, where invasion occurs (Monteville and Konkel, 2002). However, by stimulating ERK activation, *C. jejuni* not only establishes the *Campylobacter* invasion complex necessary for cell invasion but also drives the expression of cellular genes to alter a cell's behavior.

The dynamic interplay between the host and a pathogen requires that each respond to changing environments and insults. All pathogens, including *C. jejuni* (Negretti et al., 2019), express genes responsive to the conditions of a particular niche, and in turn, the host responds. We conclude that ERK1/2 is a key target that *C. jejuni* uses to potentiate infection (cell invasion and inflammation). More specifically, the experiments blocking activated ERK movement to the nucleus revealed that ERK nuclear translocation is required for *C. jejuni* to maximally invade cells. We propose that our findings are applicable to other infections, given that several pathogenic bacteria and viruses manipulate the cell cycle for replication. Identifying the genes expressed in response to early *C. jejuni* infection will provide a platform for the rational design or repurposing of U.S. Food and Drug Administration-approved host-directed therapeutics.

## Data Availability

The raw data supporting the conclusions of this article will be made available by the authors, without undue reservation.
