# Supplementary material for: Cytoplasmic and nuclear extracellular signal-regulated kinases are necessary for Campylobacter jejuni infection
Source: Front Microbiol. 2026 Jun 8;17:1854036. doi: 10.3389/fmicb.2026.1854036 (PMC13284076; doi:10.3389/fmicb.2026.1854036)
Supplement: Supplementary file 1 [file Data_Sheet_1.pdf]

Supplementary Material

Supplementary Figures

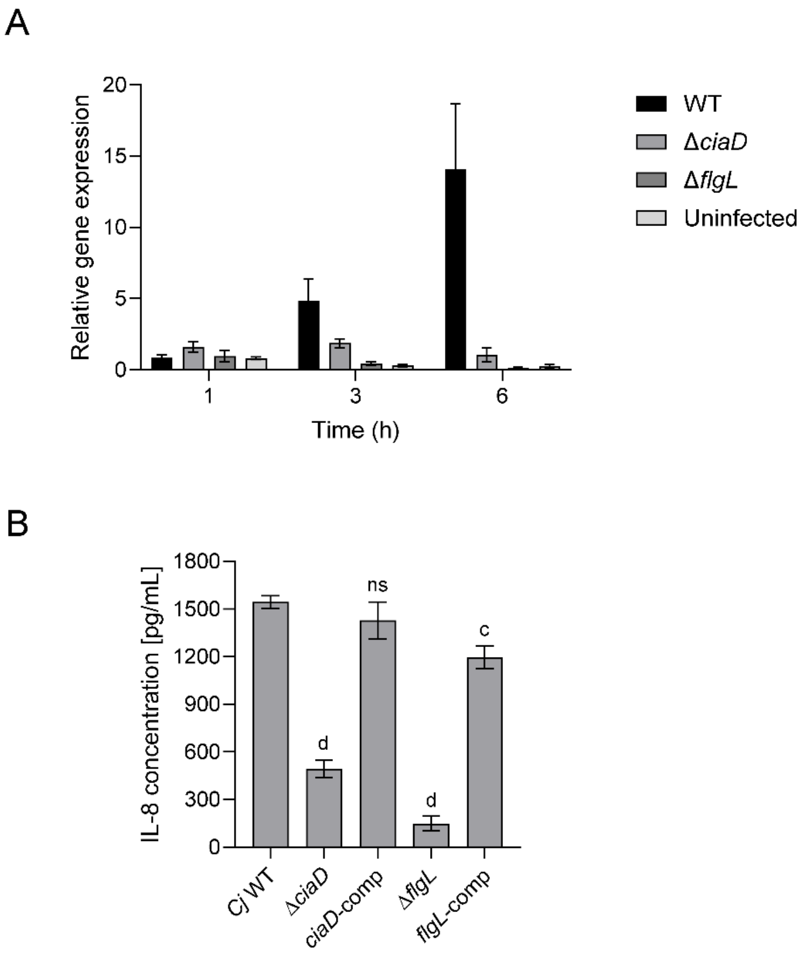

Supplementary Figure 1. *C. jejuni*-induced IL-8 secretion in INT 407 cells. IL-8 gene (*CXCL-8*) expression and IL-8 secretion in *C.jejuni*-infected INT 407 cells were determined by RT-qPCR and IL-8 ELISA, respectively. (A) INT 407 cells were infected with *C. jejuni* wild-type (WT), Δ*ciaD* mutant, and Δ*flgL* mutant isolates. Uninfected cells were used as a negative control. Total RNA was extracted from samples collected at 1 h, 3 h and 6 h post-infection and RT-qPCR was performed to determine the *CXCL-8* gene expression relative to the housekeeping gene *GAPDH*. The IL-8 gene (*CXCL-8*) expression increased at both 3 h and 6 h time points in WT-infected cells. However, no increase in gene expression occurred for either Δ*ciaD* mutant or Δ*flgL* mutant infected cells and the level of relative gene expression was comparable to that of uninfected cells. (B) The amount of IL-8 in supernatants collected from *C. jejuni*-infected cells at 24 h post-infection. Both Δ*ciaD* and Δ*flgL* mutants significantly reduced IL-8 secretion from infected cells compared to the WT strain (one-way ANOVA followed by Dunnett's multiple comparison test, c, P <0.001; d, P <0.0001; ns, not significant). The grey bars show the mean ± standard deviation from each sample (with four replicates).

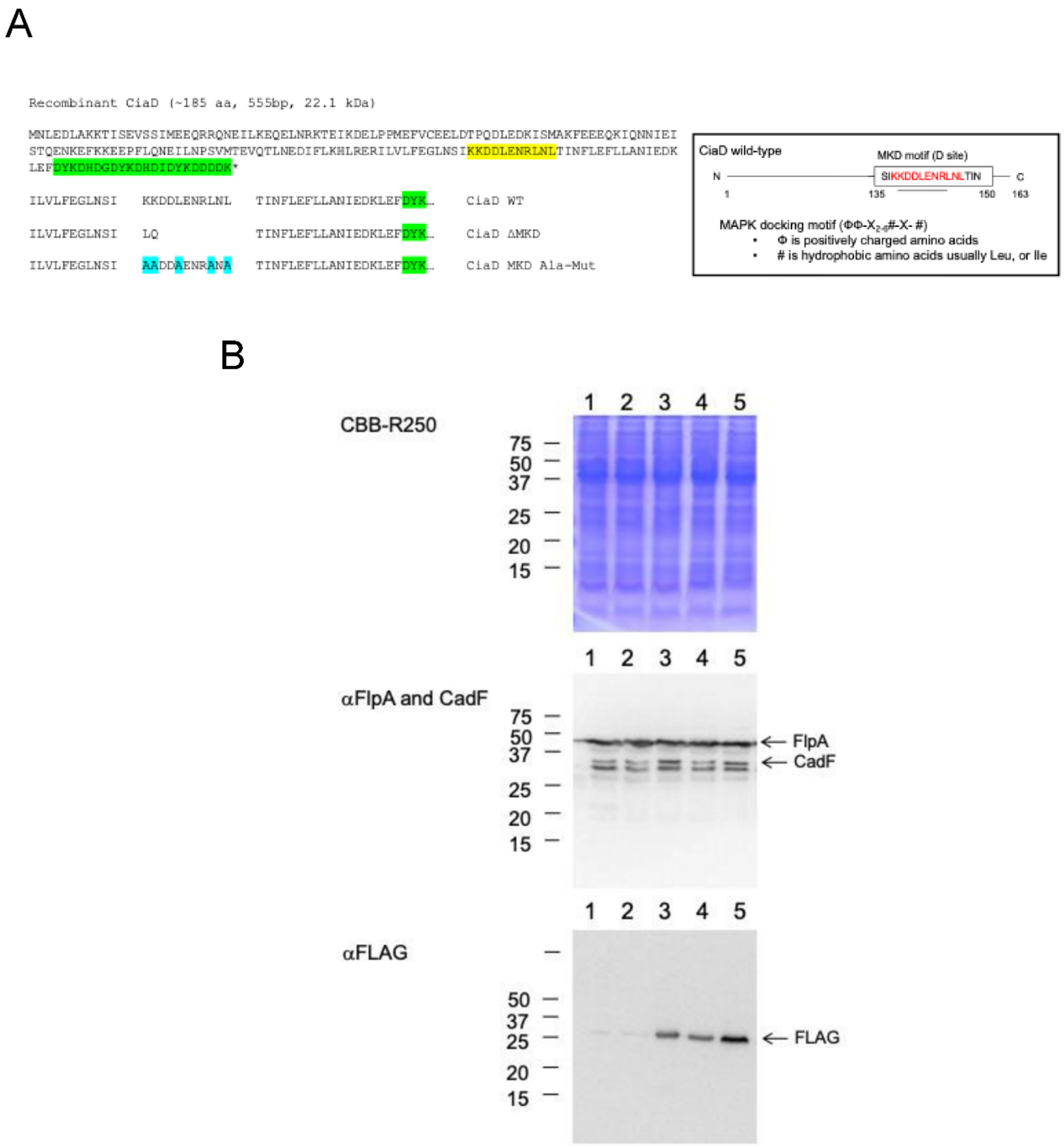

Supplementary Figure 2. Generation of CiaD MKD motif mutants. (A) Extracellular signal-regulated kinase 1/2 (ERK1/2) activation occurs through proteins containing a "mitogen-activated protein (MAP) kinase docking" (MKD) motif. A putative MKD motif in CiaD was identified using the web-based Eukaryotic Linear Motif resource. CiaD MKD motif variants were generated [MKD deletion (ΔMKD), MKD alanine mutation (MKD Ala-mut), WT MKD complement (WT MKD) in *C. jejuni* wild-type (WT) strain 81-176. All *C. jejuni* transformed isolates were confirmed by PCR amplification using gene-specific primers and sequencing of the amplified products. Yellow = MKD motif, Green = 3× FLAG tag, Cyan = MKD motif-key residues changed to alanine. (B) Whole cell lysates were prepared from the *C. jejuni* WT, Δ*ciaD* mutant, and Δ*ciaD* isolates harboring different CiaD MKD variants and analyzed by SDS-polyacrylamide gel electrophoresis and immunoblots. The following lysates were loaded into each lane: 1) WT, 2) Δ*ciaD* mutant, 3) CiaD complemented isolate (WT MKD 3× FLAG), 4) MKD alanine mutant (MKD Ala-mut-3× FLAG), and 5) MKD deletion mutant (ΔMKD-3× FLAG).

Proteins were transferred to a polyvinylidene fluoride (PVDF) membrane for immunoblot analysis. Top panel: Proteins stained with Coomassie Brilliant Blue R-250 (CBB-R250). Middle panel: Blot probed with a rabbit α-FlpA serum and α-CadF serum. Bottom panel: Blot probed with a rabbit α-FLAG antibody. Replicates of the gel were transferred to blots and probed for CadF and FlpA (unique identifiers of *C. jejuni*) and FLAG (to identify MKD variant strains). The presence of CadF and FlpA in each of the isolates confirms that they are *C. jejuni.* The presence of FLAG-tagged protein in each of the MKD variants demonstrates that the isolates synthesized the recombinant proteins. Molecular mass size standards, in kilodaltons, are indicated on the left.

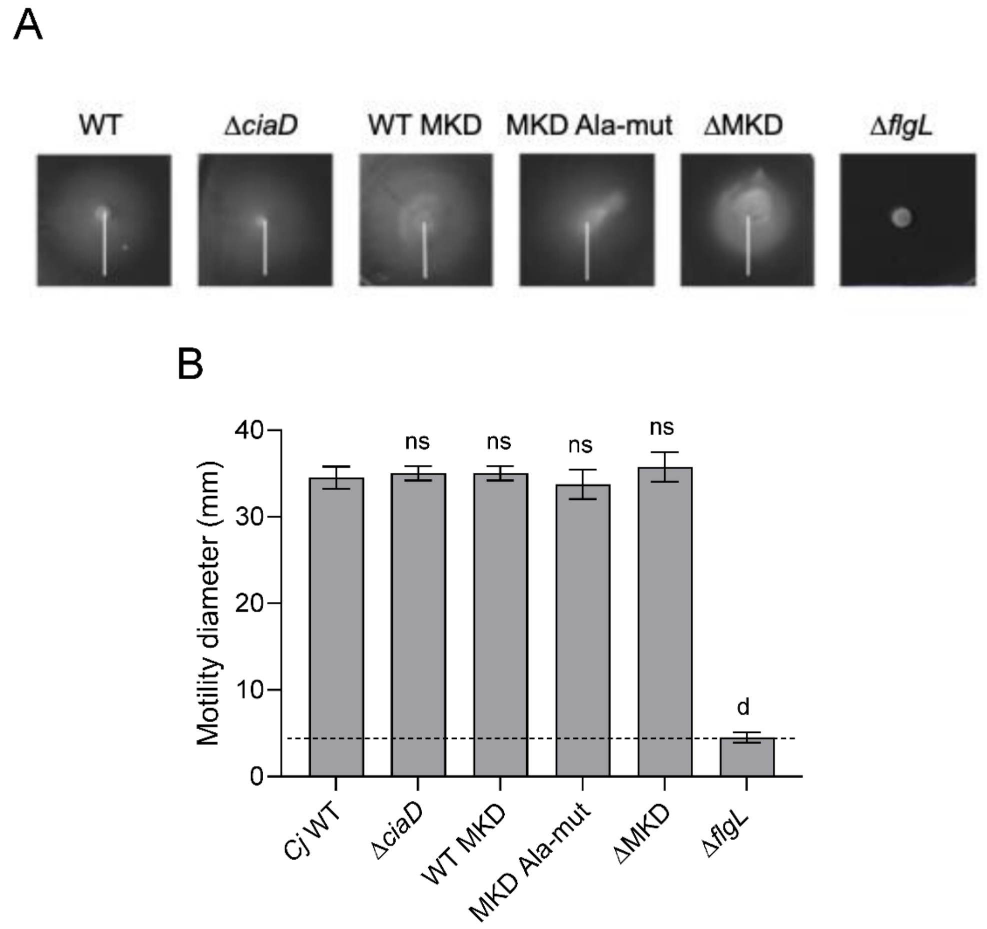

Supplementary Figure 3. The *C. jejuni* CiaD-MKD variant isolates are motile. CiaD mitogen-activated protein kinase docking (MKD) motif variants were generated [MKD deletion (ΔMKD), MKD alanine mutation (MKD Ala-mut), WT MKD complement (WT MKD) in strain 81-176. These mutants were tested alongside *C. jejuni* wild-type (*Cj* WT), Δ*ciaD* mutant and Δ*flgL* mutant isolates. Bacterial motility was tested by pipetting 3 µL of bacterial culture onto a soft agar plate followed by incubation at 37 °C for 24 h. The level of motility was determined by measuring the diameter of the zone of bacterial swarm. A) Images of bacterial swarm for different *C. jejuni* isolates on soft agar. B) The level of motility of different *C. jejuni* strains. All *C. jejuni* CiaD-MKD variants showed comparable motility to the WT strain. As expected, the Δ*flgL* mutant, a non-motile strain, presented only bacterial growth, but no bacterial swarm on soft agar. The dotted line indicates the zone of bacterial growth without motility from the spot of Δ*flgL* mutant. The grey bars present the mean ± standard deviations from the measurements of four replicates. Statistical significance was calculated by one-way ANOVA followed by Dunnett's multiple comparison test. d, $P < 0.0001$; ns, not significant ($P > 0.05$).

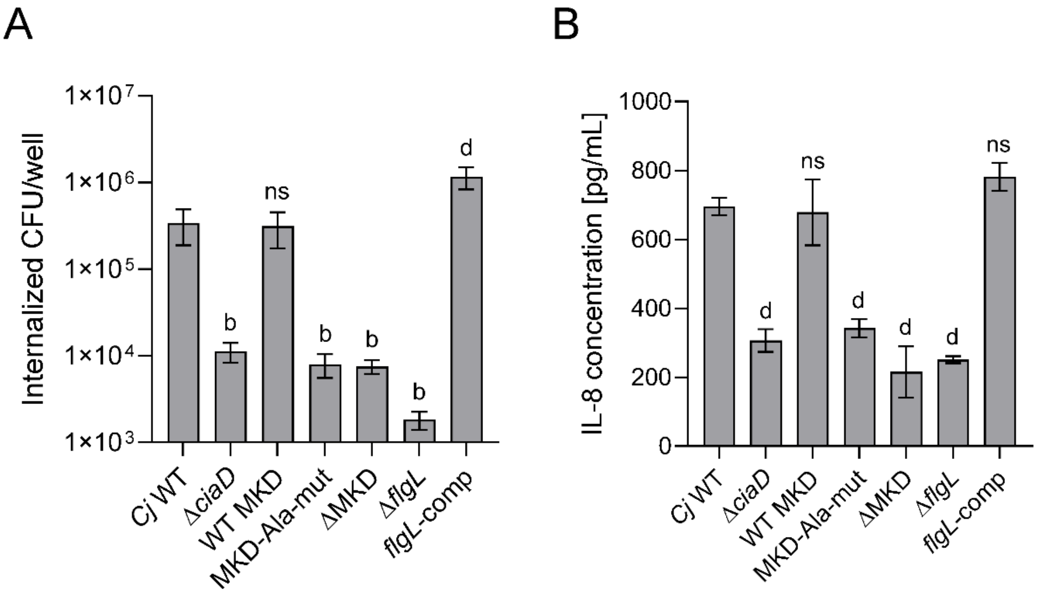

Supplementary Figure 4. *C. jejuni* CiaD-MKD variant isolates are impaired in INT 407 cell invasion and IL-8 secretion. INT 407 cells were infected with a *C. jejuni* 81-176 wild-type (*Cj* WT) strain, Δ*ciaD* mutant, *ciaD* complement (WT MKD), CiaD MKD alanine mutant (MKD-Ala-mut), CiaD MKD deletion mutant (ΔMKD), Δ*flgL* mutant, and *flgL* complement (*flgL*-comp) isolates, and *C. jejuni* internalization and IL-8 secretion were measured by the gentamicin-protection assay and ELISA, respectively. A) The number of internalized *C. jejuni* in INT 407 cells. A Δ*flgL* mutant (non-motile strain) was used as a baseline for internalized bacteria. MKD Ala-mut and ΔMKD isolates showed a significant reduction in bacterial internalization in cells compared to the WT strain (one-way ANOVA followed by Dunnett's multiple comparison test, b, $P<0.01$; d, $P<0.0001$; ns, not significant). B) The amount of IL-8 in supernatants collected from *C. jejuni*-infected cells at 3 h post-infection. MKD Ala-mut and ΔMKD isolates significantly reduced IL-8 secretion from infected cells compared to the *C. jejuni* WT strain (one-way ANOVA followed by Dunnett's multiple comparison test, d, $P<0.0001$; ns, not significant). The grey bars represent mean ± standard deviations from the measurements of four replicates.

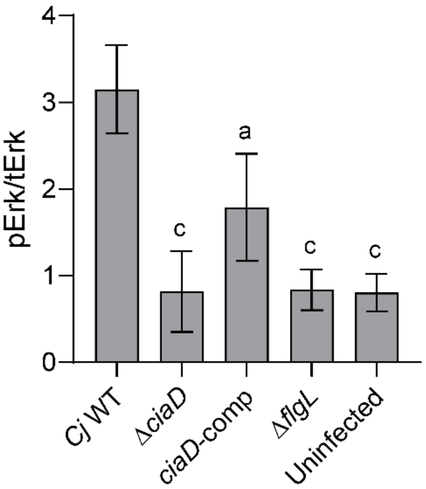

Supplementary Figure 5. A *C. jejuni* Δ*ciaD* mutant is impaired in ERK1/2 activation. INT 407 cells were infected with either a *C. jejuni* wild-type strain (*Cj* WT), *ciaD* mutant (Δ*ciaD*), *ciaD* complement (*ciaD*-comp), *flgL* mutant (Δ*flgL*), or left uninfected. Cell lysates were collected from *C. jejuni*-infected cells after a 75-min infection period. The protein concentration of total ERK1/2 (tERK) and phosphorylated ERK1/2 (pERK) was measured using an ERK1/ERK2 (Total/Phospho) ELISA. The ratio of pERK to tERK was calculated for each *C. jejuni* isolate. Cells infected with the Δ*ciaD* mutant had a pERK/tERK ratio of 0.87, comparable to that of the negative controls (Δ*flgL* and uninfected) and significantly less than the WT strain (one-way ANOVA followed by Dunnett's multiple comparison test, a, $P < 0.05$; c, $P < 0.001$). The mean ± standard deviations from each sample (with four replicates) are shown in a bar graph.

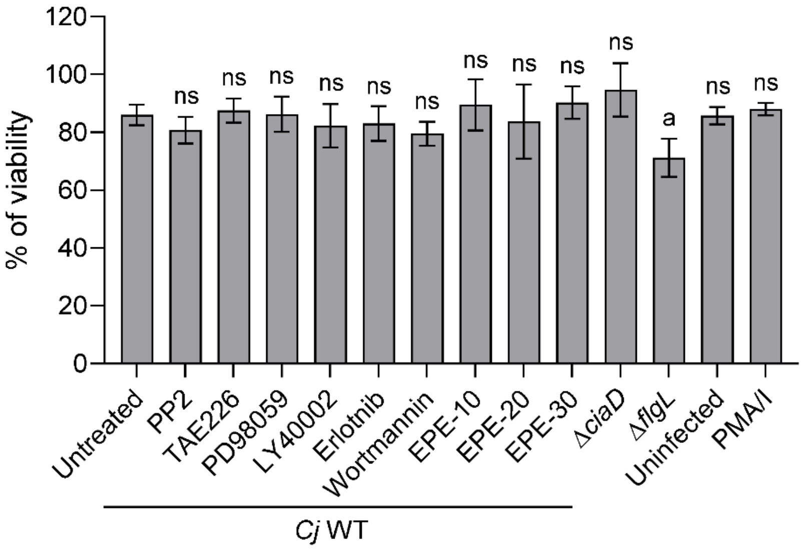

Supplementary Figure 6. Viability of INT 407 cells with inhibitors targeting focal adhesions and Raf/MEK/ERK MAPK signaling. Cell viability was assessed by the trypan blue exclusion assay as described in the materials and methods. Results are presented as the percent cell viability of *C. jejuni*-infected and uninfected cells in the presence of inhibitors. Significant differences in values compared to untreated samples were determined by one-way ANOVA followed by Dunnett's multiple comparison test. a, $P < 0.05$; d, $P < 0.0001$, ns, not significant ($P > 0.05$).

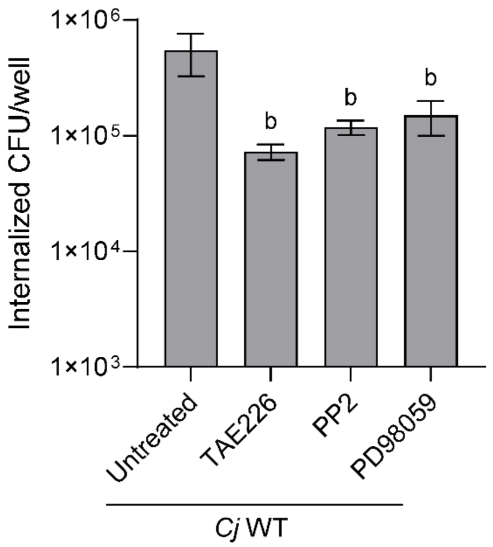

Supplementary Figure 7. The effect of focal adhesion and MEK/ERK signaling pathway inhibitors on *C. jejuni* cell invasion. INT 407 cells were infected for 3 h with a *C. jejuni* wild-type strain in the absence and presence of one of the three inhibitors: TAE 226 (FAK inhibitor), PP2 (c-Src inhibitor), and PD98059 (ERK 1/2 activation inhibitor), and *C. jejuni* internalization was measured by the gentamicin-protection assay. The plot shows the number of internalized *C. jejuni* in treated and untreated INT 407 cells. There was a significant reduction in *C. jejuni* internalization in treated cells compared to the untreated cells (one-way ANOVA followed by Dunnett's multiple comparison test, b, $P < 0.01$). The grey bars represent mean ± standard deviation from the measurement of four replicates.
